# Thermal baths as sources of pharmaceutical and illicit drug contamination

**DOI:** 10.1007/s11356-019-06633-6

**Published:** 2019-12-02

**Authors:** Gergely Jakab, Zoltán Szalai, Gábor Michalkó, Marianna Ringer, Tibor Filep, Lili Szabó, Gábor Maász, Zsolt Pirger, Árpád Ferincz, Ádám Staszny, Péter Dobosy, Attila Csaba Kondor

**Affiliations:** 1 Geographical Institute, Research Centre for Astronomy and Earth Sciences, Hungarian Academy of Sciences, Budaörsi út 45, Budapest, H-1112 Hungary; 2 Department of Environmental and Landscape Geography, Eötvös Loránd University, Pázmány Péter sétány 1/C, Budapest, H-1117 Hungary; 3 Institute of Geography and Geoinformatics, University of Miskolc, Egyetemváros, Miskolc, H-3515 Hungary; 4 MTA-Centre for Ecological Research, Balaton Limnological Institute, Klebelsberg Kuno u. 3., Tihany, H-8237 Hungary; 5 Department of Aquaculture, Szent István University, Páter K. u. 1, Gödöllő, H-2100 Hungary; 6 MTA-Centre for Ecological Research, Danube Research Institute, Karolina út 29, Budapest, H-1113 Hungary; 7 Corvinus University of Budapest, Fővám tér 8, Budapest, H-1093 Hungary

**Keywords:** Discharged thermal wastewater (DTWW), Surface water contamination, Pharmaceutically active compounds (PhACs), Tourism

## Abstract

Despite the fact that there are tens of thousands of thermal baths in existence, knowledge about the occurrence of pharmaceutically active compounds (PhACs) in untreated thermal wastewater is very limited. Because used thermal water is typically legally discharged into surface waters without any treatment, the effluent poses environmental risks for the receiving water bodies. The aim of this study was to show the occurrence patterns and spatiotemporal characteristics of 111 PhACs in thermal wastewater. Six thermal water outflows of different thermal baths were tested in different seasons in the Budapest metropolitan region (Hungary), and diurnal analysis was performed. After solid-phase extraction, the samples were analysed and quantified by coupling supercritical fluid chromatography and mass spectrometry to perform simultaneous multi-residue drug analysis. The results confirm that water discharge pipes directly transport pharmaceuticals into surface water bodies; 34 PhACs were measured to be over the limit of quantification at least once, and 21 of them were found in more than one water sample. The local anaesthetic drug lidocaine, antiepileptic carbamazepine, analgesic derivative tramadol and illicit drug cocaine were detected in more than half of the samples. Caffeine, metoprolol and bisoprolol (cardiovascular drugs), benzoylecgonine (cocaine metabolite), diclofenac (NSAID), citalopram (antidepressant) and certain types of hormones also have a significant frequency of 30-50%. However, the occurrence and concentrations of PhACs vary according to the season and number/types of visitors. As demonstrated by the diurnal fluctuation, drug contamination of thermal waters can significantly vary, even for similar types of baths; furthermore, the quantity and types of some pollutants rapidly change in the discharged thermal wastewater.

**Electronic supplementary material:**

The online version of this article (10.1007/s11356-019-06633-6) contains supplementary material, which is available to authorized users.

## Introduction

Surface waters are polluted by pharmaceutically active compounds (PhACs), which are regarded as widespread contaminants (Aus der Beek et al. 2016; Daughton and Ternes 1999; Deo 2014; Kümmerer 2008; Li et al. 2019). The negative impact of certain PhACs, such as endocrine-disrupting chemicals (e.g. hormones), antidepressants, sedatives, anaesthetics, recreational substances or illicit drugs, on aquatic ecosystems has been proven in laboratories and in nature (Bókony et al. 2018; Capaldo et al. 2018; Maász et al. 2017; Martin et al. 2017). This problem is exacerbated by the fact that some of the more persistent and slowly decomposing agents reach the drinking water supply (Leung et al. 2014; Tröger et al. 2018) and are absorbed by plants through irrigation (Malchi et al. 2014; Margenat et al. 2019). These PhACs consequently appear in the human food chain (Carter et al. 2014), even though their concentration is rather low.

The European Union has referred to the Water Framework Directive to establish a watchlist of the most important contaminants that need to be monitored. The list was last updated in 2018, and it includes several PhACs, as among others oestrone (E1), 17β-estradiol (E2), 17α-ethinylestradiol (EE2), diclofenac and macrolides (EU 2018), the sources of which will have to be identified, monitored and screened under more scrutiny in the future (Castiglioni et al. 2018; Könemann et al. 2018).

In addition to communal sewage (Kasprzyk-Hordern et al. 2008; König et al. 2017; Roberts and Thomas 2006), PhACs can contaminate the environment via other legal sources, such as grey waters used for irrigation (Etchepare and van der Hoek 2015; Lees et al. 2016). Thermal spa water that has been discharged into natural waters is also considered to be a legal source of contamination. Thermal water used for bathing, unlike that utilized for purposes of energetics, must not be reinjected into the aquifer because of the presence of bacteria and other contaminants, therefore it is typically discharged into surface receivers. Although, in general, used thermal water is known to have a potentially harmful environmental impact (e.g. heat and salt load; Benz et al. 2017; Farsang et al. 2015; Kiss et al. 2013), little remains to be known about the level of their pharmaceutical contamination, as there are few reports on PhAC contamination of used thermal water-sourced surface water. A related test was carried out by Avar et al. (2016a, b); it revealed the existence of EE2 (0.52 ng L^−1^) and other hormones (drospirenone, levonorgestrel, progesterone; 1.26-2.28 ng L^−1^) in the Hévíz-Páhoki Canal, which is fed by Lake Hévíz, one of the largest thermal lakes in the world. Additionally, the findings of Mackuľak et al. (2014, 2016) in the spa town of Piešt’any, Slovakia indicate that a higher than average presence of illicit drugs and anaesthetics (e.g. tramadol) should be expected.

The global utilization of thermal water is increasing in coincidence with increasing health and wellness tourism (Smith and Puczkó 2014). Figures released by the Global Wellness Institute show the extent to which the thermal mineral springs industry contributes to the more than 4.2 trillion USD wellness economy, with approximately 34,000 establishments; this industry slightly overlaps the more generalized spa industry, which has 150,000 establishments (Global Wellness Economy Monitor 2018). Thermal spas that use water from hot springs or drilled wells can be found in nearly 130 countries. Several thousand establishments discharge untreated thermal water into natural receivers, thereby harming the vulnerable ecosystem.

Earlier research has proven that a significant amount of PhACs enter swimming pool water during use. Most of this contamination is the result of unhygienic behaviour (e.g. urination, defecation, gargling, vomiting) or the rinsing of chemicals (e.g. creams, plasters) off of the skin (Ekowati et al. 2016; Fantuzzi et al. 2018; Lindsay et al. 2017). Other bodily fluids, such as perspiration due to warm water, can also play an important role (Kanan and Karanfil 2011; Keuten et al. 2014). To date, PhAC monitoring has mainly been performed using the water of swimming pools with water recirculation technology, and where the water is disinfected with chlorine and undergoes further treatment before being partially discharged into the communal sewage system; thus, this water does not reach natural waters directly. Ekowati et al. (2016) sampled 17 Catalonian pools, and 10 of the 32 monitored PhACs exceeded the limit of quantification (LOQ) value; particularly, carbamazepine was found to be ubiquitous (27 of 51 water samples). Fantuzzi et al. (2018) tested the occurrence of illicit drugs; they found some of their metabolites and 48 pharmaceuticals in 10 indoor swimming pools in Italy. They also found 11 of the 48 monitored PhACs; regarding illicit drugs, only cocaine and its metabolites were identified in nine swimming pools.

Disinfection and chlorination in swimming pools help to keep certain pharmaceutical substances (e.g. naproxen, acetaminophen; Weng et al. 2014) at an undetectable level; however, the reactive chlorine may induce the creation of metabolites that can be more toxic than the original compound (Judd and Bullock 2003; Kanan and Karanfil 2011; Richardson et al. 2010; Teo et al. 2016; Yue et al. 2016). Alternatively, water recirculation technology can also influence the amount of certain PhACs, as some of the water remains in the system for longer time periods, i.e. up to a few weeks, therefore allowing chlorine-resistant compounds to accumulate (Ekowati et al. 2016; Fantuzzi et al. 2018). In the case of swimming pools, the incoming tap water may already be contaminated by PhACs (Suppes et al. 2017). However, in the case of thermal spas, the filling water is typically sourced from hundreds of metres underground, is above 30 °C, has high mineral content and is free from anthropogenic contamination. Thus, to preserve its therapeutic effects, the water cannot be diluted with municipal water and cooled, and it cannot be disinfected like the water of swimming pools, therefore, there is a larger amount and variety of active microbial life in thermal water as compared to treated water. The high biological activity can breakdown organic molecules (even PhACs), and a number of metabolites can be created (Szuróczki et al. 2016). Thermal pools typically have a filling and draining system, or an instantaneous system. The used thermal water is continuously and directly discharged into natural waters without any further treatment; this means that the PhACs that it may contain are also discharged into natural waters (Farsang et al. 2015; Kim 1999). In countries in temperate and cold zones, where most thermal spas can be found, spa use is more seasonal than swimming pool use, and this impacts the potential contamination of the outflowing water (CP 2015, Ferrante et al. 2018, Duro and Turrión-Prats 2019). Tourist influx in the summer causes the number of visitors to increase, and can also profoundly impact contamination levels. Based on statistics the types of winter and summer visitors significantly differ (Csapó and Marton 2017). Tourists from abroad are overrepresented in summer visitors, whereas the ratio of elderly locals is higher during the winter (HCSO 2017). Thus, it is necessary to determine the effects of wellness and therapeutic tourism on PhAC loads in thermal spas.

Hungary, particularly its capital, Budapest, has a number of thermal spas, some of which are internationally renown (Erfurt-Cooper and Cooper 2009). In terms of the number of thermal springs and the overall industry, the country has a high global ranking (Global Wellness Economy Monitor 2018; Michalkó and Rácz 2010). Although there are risks attributed to the presence of PhACs and illicit drugs in surface waters, and there are many thermal bath outflows all over the world, the contribution of thermal spas has not yet been investigated. Therefore, in this study, the concentrations of PhACs in discharged thermal wastewater (DTWW) were investigated by using Hungarian examples. The analysis summarized in this paper was performed within the framework of a 3-year-long research project supported by the Hungarian government that examined PhAC contamination in the Budapest metropolitan region.

The aim of the study was i) to determine which PhACs can be detected in DTWW; ii) to determine if the levels of PhACs differ between the internationally recognized spas frequented by tourists (‘international baths’), and the baths that mainly attract local inhabitants (‘local baths’); iii) to determine if the above-mentioned differences vary according to the season; and iv) to determine if the levels of PhACs in internationally recognized baths fluctuate within one single day in the high-tourist season.

## Materials and methods

### Sampling properties

Water samples were collected from the open-ended water discharge pipes of six Hungarian thermal baths in and around Budapest from which effluent is directly transported to surface waters. Each of these baths have drilled thermal wells, and the sampled water outflows were located 10-20 m from the baths. In this study, the baths were blindly marked as A through F. Spas A, B and C are located in the central part of Budapest; their number of visitors exceeds 300,000 per year. They are open to tourists throughout the entire year, and, in addition to the pools with certified therapeutic water, they also offer cold-water pools for recreational purposes. Spas D, E and F are located in the outskirts and suburbs of Budapest. They are also open throughout the entire year, and, like Spas A-C, they have pools with therapeutic water, and cold-water pools. However, these spas are smaller, and receive 150,000-300,000 visitors per year (HCSO 2017). The thermal pools of the sampled spas are visited by more than 100 people in a single day in the winter, at the larger spas, the number of thermal pool users exceeds 1000 people per day in summer.

The sampled water pipes directly transport the used thermal water collected from the thermal pools to surface waters. The water from the discharge pipes is not directly related to the nominal capacity of thermal wells, and some thermal spas have more than one water outflow. The volume of the DTWW significantly fluctuates; specifically, at peak times, it is typically 100-200 L min^−1^, which can vary depending on the operations of the bath. The fluctuation of the temperature of the effluent was minimal (28-35 °C), regardless of the season or the establishment; this is because the water temperature of the pools designated for therapeutic purposes ranges from 30 to 35 °C, and the water is directly transported to the water discharge pipe without any further dilution or cooling. Overall, the water chemistry-related parameters (pH, conductivity, mineral content) of the sampled water were consistent with the official data on the certified thermal waters, as provided by each of the spas; therefore, the volume of non-thermal-pool water in outflow pipes, such as water sourced from non-thermal pools, was, with one exception, negligible at the time of sampling.

To examine seasonal fluctuation, the samples were collected in the off-season (15 February 2018, Thursday), pre-season (10 June 2018, Sunday) and main tourist season (26 July 2018, Thursday), this corresponds to 6 spas × 1 sample × 3 seasons = 18 samples. The sampling was always performed between the time period of 13:00 and 16:00, as the contaminated thermal water was presumed to be passing through the discharge pipes by this time because of the filling and draining system. However, the off-season sample from Spa B was corrupted during laboratory preparations, causing the measured values to be unreliable; therefore, they were not used in the analysis. Thus, 17 water samples were used in the seasonal analysis. To examine diurnal PhAC content fluctuation in the DTWW, Spas A and B were sampled every 3-4 h. It was not feasible to sample Spas A and B on the same day because of logistical problems, namely - parallel with diurnal monitoring at Spa A - other spas were also sampled. Therefore, diurnal samples were obtained from Spa B during a large-scale international music festival. Because the admission fee to the festival included free access to the spa, there was a large number of foreign visitors. A total of seven (26 July 2018, Thursday, 6-24 h) and four (12 August 2018, Sunday, 8-20 h) samples were collected from Spas A and B, respectively; note that an (unplanned) additional sample was obtained from Spa A because the composition of the discharged water was visibly observed to suddenly change. Thus, diurnal fluctuations were analysed as based on 12 samples from two locations. Spa B has two thermal water outflows, and the water from the other discharge pipe is used by a different institution for heating and irrigation; therefore, the sampled outdoor outflow was not always operating at full capacity. Consequently, four samples were collected per day, at times when the output water pressure was high for longer periods of time.

All samples (2.5 L for PhACs) were collected in amber silanised glass bottles with Teflon faced caps (Thermo Fisher Scientific) as grab samples and transported to the laboratory in a dark cooler filled with ice within 4 h. The samples were stored in a dark environment at 4 °C and extracted within 20 h, thereby, the sample was fully prepared within 24 h from the sampling. The evaporated samples were stored at −80 °C and analysed within 30 days.

The water was sampled at the joint, open-ended water discharge pipes of the selected thermal spas; thus, the samples reflect all the thermal water pools of each spa. The differences between the pools, and those caused by the water recirculation and/or filling and draining technology, were not considered; this is because this study focused on the contamination level of the water entering the surface water. The samples collected to measure the levels of organic PhACs were preserved in formic acid at a pH level below 2.0. To determine the basic hydrochemical properties, 0.5 L of water was collected in a sterile glass container. To assess carbon and nitrogen content, 50 ml of each sample was collected and preserved in formic acid at a pH level below 2.0. To determine the heavy metal content, 15 mL of water was collected in a centrifuge tube; then, the sample was filtered by using a 0.45-μm PVA filter, and nitric acid was added until the pH level decreased to below 2.0. The temperature, conductivity and redox potential of the outflowing water were simultaneously measured as each sample was collected. All of these variables were also measured in the laboratory, Table S1 provides the corresponding values.

### Evaluation of hydrochemical properties

Dissolved carbon and nitrogen content was determined with a MULTI N/C 3100 type, Analytik Jena AG made TOC/TN instrument. The concentration of cations (ammonium, calcium, magnesium, sodium, potassium) and anions (fluoride, chloride, sulphite, bromide, nitrite, nitrate) was established with the help of a dual channel Dionex ICS 5000+ ion chromatograph. The nitrate and phosphate content of water samples was measured with a HACH DR/2000 type spectrophotometer, and its heavy metal content with a PlasmaQuant MS Elite, Analytik Jena, Jena, Germany (ICP-MS) mass spectrometer.

### PhAC analysis

Details of the sample preparation process and setup for analysis have been previously reported (Maasz et al. 2019). To summarize, the water samples were acidified with formic acid and spiked with corresponding mass-labelled internal standard to the sample quantification and compensation the matrix effect and chemical losses during the sample preparation. Due to the relatively low concentration, analytes in the filtered samples were isolated using solid-phase extraction applying Strata X-CW cartridges (33 μm, 200 mg 6 mL^−1^, #8B-S035-FCH, Phenomenex) and then eluted with ammonium hydroxide-acetonitrile solution by AutoTrace 280 automatic SPE system (Thermo Scientific). The sample was fully prepared within 24 h from the sampling. The evaporated (by nitrogen gas stream) eluates were reconstituted with acetonitrile and transferred to vials within 30 days. Derivatization (by dansyl-chloride) of steroid agents was performed to reach the appropriate sensitivity. The selected PhACs were analysed and quantified using supercritical fluid chromatography (ACQUITY UPC2 system, Waters) coupled with tandem mass spectrometry (MS) (Xevo TQ-S Triple Quadrupole, Waters). Data were recorded in three technical replicates by MassLynx software (V4.1 SCN950) and evaluated by TargetLynx XS software. Separation of compounds was performed on a 3.0 mm × 100.0 mm, 1.7 μm particle size, ACQUITY UPC2 BEH analytical column (#186007607, Waters). The MS measurement was performed in positive ion mode. The electrospray ionization source was operated at a spray voltage of 3 kV in both positive and negative ion modes, and at a cone voltage of 30 V. MS/MS experiments were performed by applying the multiple-reaction monitoring method with an isolation window of 0.4 m/z. The observed ions (mass in m/z) were accepted and quantified if the following variables were within their respective limits: MS1 mass, retention time, MS2 masses, fragmentation pattern and IS correction. Method characteristics, LOD, LOQ and validation values are listed in Table S2.

The samples were used to identify 111 PhACs, including pharmaceutical derivatives, illicit drugs and alkaloids such as cocaine and caffeine. The agents to be analysed were determined based on Hungarian consumption data and the toxicological effect profile. The PhACs were categorized into the following nine groups for analysis: 1) antidepressants, 2) antiepileptics, 3) anxiolytics, 4) cardiovascular drugs, 5) hormones and derivatives, 6) stimulants, psychedelics, hallucinogens and their metabolites, 7) nonsteroidal anti-inflammatory drugs (NSAIDs), 8) anaesthetics and analgesics, 9) other (including alkaloids, such as caffeine). The groups can be directly compared to the classification systems described in the relevant literature on PhAC contamination of swimming pools (e.g. Fantuzzi et al. 2018).

## Results and discussion

### General results

Thirty-four of the monitored 111 PhACs were found to exceed their respective LOQ value at least once in one of the water samples (Table S3); additionally, 21 of the PhACs were detected in more than one sample. There are significant differences in the frequency of occurrence and concentration levels of the detected PhACs (Table 1).

**Table 1:** Concentrations of all PhACs found to exceed their LOQ value (MIN: measured minimum value, MAX: measured maximum value, Mean: average of the measured values >LOQ)

PhACs	Pharmacological classification	Frequency of occurrence		LOQ	MIN	MAX	Mean
		Number	%			ng L^−1^	
lidocaine	anaesthetics	22	79	0.10	0.81	132.86	29.84
tramadol	analgesics	16	57	0.10	0.22	14.96	2.11
carbamazepine	antiepileptics	17	61	0.10	0.17	188.57	32.13
lamotrigine	antiepileptics	6	21	5.00	18.49	96.59	54.07
bupropion	antidepressants	1	4	0.50	na	1.16	na
citalopram	antidepressants	11	39	0.10	0.10	3.26	1.56
tiapride	antidepressants	1	4	0.10	na	0.25	na
trazodone	antidepressants	1	4	0.05	na	0.21	na
alprazolam	anxiolytics	1	4	0.10	na	0.54	na
cinolazepam	anxiolytics	1	4	0.10	na	0.36	na
betaxolol	cardiovascular drugs	1	4	0.50	1.12	1.12	0.00
bisoprolol	cardiovascular drugs	10	36	0.50	0.74	13.27	3.48
metoprolol	cardiovascular drugs	9	32	0.10	0.60	9.54	4.07
perindopril	cardiovascular drugs	4	14	0.10	0.24	0.89	0.52
propafenone	cardiovascular drugs	2	7	0.50	0.91	1.55	1.23
verapamil	cardiovascular drugs	1	4	0.05	na	0.56	na
benzoylecgonine	stimulants (metabolite)	9	32	0.10	0.67	6.47	2.93
cocaine	stimulants	15	54	0.05	0.14	194.02	30.33
ketamine	hallucinogenic drugs	1	4	0.50	na	57.00	na
norketamine	hallucinogenic drugs	1	4	5.00	na	10.37	na
oestrone	hormones	12	43	0.05	0.10	112.59	12.03
17α-estradiol	hormones	10	36	0.05	0.05	39.48	4.42
17β-estradiol	hormones	1	4	0.05	na	5.60	na
estriol	hormones	7	25	0.05	0.07	2.09	0.58
17α-etynylestradiol	hormones	13	46	0.05	0.64	98.33	17.22
testosterone	hormones	7	25	0.50	0.61	97.31	22.51
progesterone	hormones	9	32	0.50	0.51	10.24	2.98
levonorgestrel	hormones	3	10	1.00	1.06	8.19	3.70
drospirenone	hormones	1	4	1.00	na	1.84	na
paracetamol	NSAIDs	1	4	20.00	na	76.10	na
diclofenac	NSAIDs	12	43	0.50	1.61	57.59	24.33
theophylline	other (alkaloids)	6	21	10.00	59.43	7184.16	3308.93
caffeine	other (alkaloids)	9	32	10.00	484.96	2061.43	1347.20
papaverine	other (alkaloids)	1	4	0.10	na	1.36	na

Theophylline was found to have the highest absolute concentration (max = 7184 ng L^−1^); however, those were frequent only at a rate of 20%. The average concentration of caffeine, i.e. the most common stimulant, exceeded 1000 ng L^−1^. It is present in various food items and health supplements (e.g. coffee, energy drinks) as a natural component, and is not related to the consumption of pharmaceuticals; however, it can be an indicator that the spa water has been contaminated by urine and/or other bodily fluids (Teo et al. 2016). As was observed with two antiepileptics (carbamazepine and lamotrigine), three types of hormones (E1, EE2 and testosterone) and the illicit drug cocaine, lidocaine (anaesthetic) and diclofenac (NSAID) the latter is an agent of several non-prescription drugs, were found to have an average concentration above 10 ng L^−1^. Note that, whether a PhAC is prescription or non-prescription does not impact the frequency of its occurrence in the samples. For some active substances (e.g. carbamazepine, diclofenac, cocaine), the results were generally consistent with what was expected as based on the results for swimming pools or different types of polluted waters frequented by tourists; however, until this study, most of the above-mentioned PhACs had not been measured in bathing waters. It should be noted that 17α-estradiol (aE2) and E2, which are generally detected in all types of environmental monitoring assessments (Aus der Beek et al. 2016), were detected, but the frequency of detection of the former was much higher in the sampled thermal spa water.

**Fig. 1:**
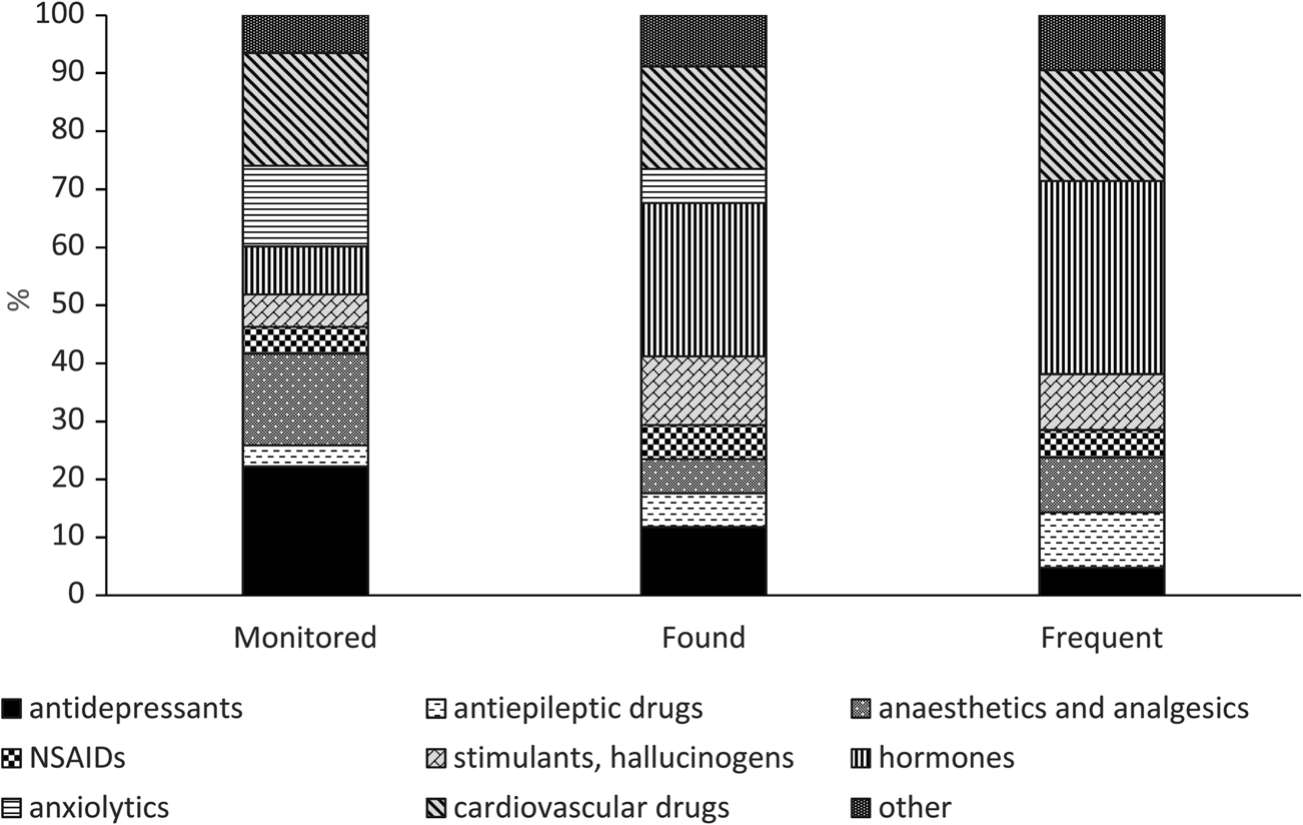
PhACs compositions in the Monitored (all 111 PhACs); Found (detected in at least one sample, >LOQ) and Frequent (detected in more than one sample > LOQ) groups

These findings can be used to compare the proportions of the different groups of PhACs (Fig. 1). Although hormones were the most frequent, the occurrence of hallucinogenic drugs was also higher than their ratio within then monitored 111 PhACs. The increased proportion of antiepileptics (e.g. carbamazepine, lamotrigine) is also significant; conversely, the proportion of antidepressants decreased in the found and frequent groups. Moreover, as with swimming pool water (Ekowati et al. 2016; Fantuzzi et al. 2018), this group of PhACs, particularly carbamazepine, was most frequently found in thermal spa water. In contrast, anxiolytics and anaesthetics were not observed in high concentrations, although some of their representative compounds, such as tramadol and lidocaine, were detected in many of the samples. The latter two PhACs were also found to be highly persistent, as reported by Bollmann et al. (2016), Wood et al. (2017), Malchi et al. (2014) and López-García et al. (2018). It should be noted that neither the occurrence or concentration of PhACs was found to be related to the chemical properties of the spa water (Table S1). This also suggests that PhAC content is independent of the water source, and that the analysed PhACs are chemically stable enough to not interact with the high solute components of the thermal water.

Regarding the PhACs that were detected only once, Spa B, which is very popular with foreign visitors, exhibited the highest rate of occurrence of single detection (four of six samples). These PhACs include antidepressants (trazodone), NSAIDs (paracetamol) and hallucinogenic drugs (ketamine and norketamine). Accordingly, the lowest occurrence (three of 10 samples) was found at Spa A, which also has a high number of domestic and international visitors; one antidepressant (bupropion) and two cardiovascular drugs (betaxolol, verapamil) were detected only once there. Alternatively, one antidepressant (tiapride) and one anxiolytic (cinolazepam) were found to be unique agents in three samples from Spa C. For the local spas, there was no single occurrence at Spa D, and, in the case of Spas E and F, the single-occurrence PhACs were in the group termed ‘other’, e.g. papaverine and anxiolytics (alprazolam, cinolazepam). Although there were single occurrences of antidepressants at all of the international baths, this was not the case for any of the local spas. Note that the above-mentioned PhACs were not included in further analysis since they were only found in one sample.

### Seasonal and geographical analysis

**Fig. 2:**
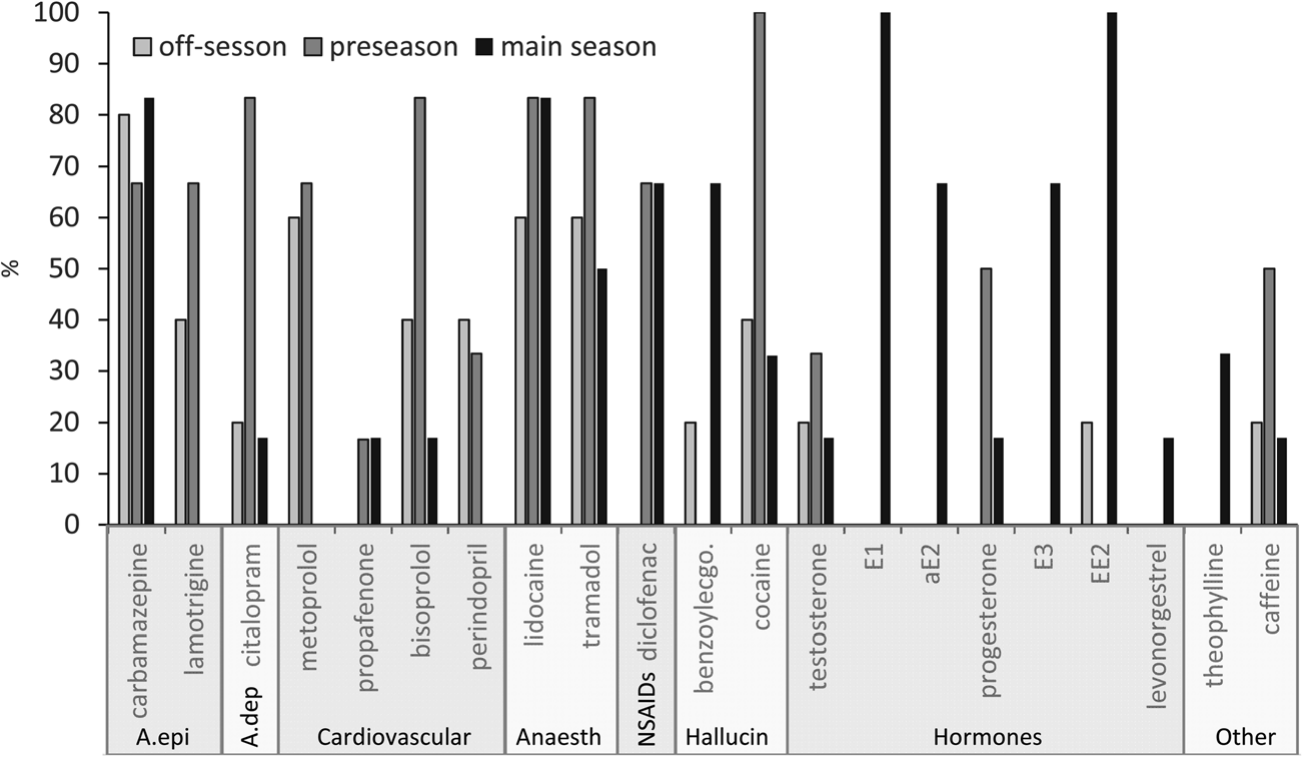
Seasonal occurrence frequency of the detected drugs (%); A.epi: antiepilepticum; A.dep: antidepressants; Cardiovascular: cardiovascular drugs; Anaesth: anaesthetics and analgesics; NSAIDs: nonsteroidal anti-inflammatory drugs; Hallucin: stimulants, hallucinogens and their metabolites

Seasonal analysis of the 17 samples collected from all outflows revealed hormones to be the most prevalent group in the summer. Of the eight detected hormones, only testosterone was found to occur in every season; hormones related to contraceptives were detected in all of the summer samples (Fig. 2, Table S3). This is consistent with the empirical fact that young women tend to visit thermal baths more often during their summer holiday (HCSO 2017).

Alternatively, drugs used for the treatment of cardiovascular disorders (e.g. bisoprolol, metoprolol, perindopril) were most prevalent in the tourism off-season. Two of the four cardiovascular PhACs were not detected in the high-tourist season, and the remaining two were only found in a few samples. As a hypothesis, this may indicate that older generations prefer to visit thermal spas in the off-season, as it was also reported by Löke et al. (2018).

Various PhACs, such as the anaesthetics tramadol and lidocaine, were found in most of the water samples, regardless of the season. The possible reasons for the high proportion and persistence of antiepileptics have been discussed above. It should be noted that the absence of lamotrigine in the summer samples was unexpected. This phenomenon cannot be explained using the currently available results; thus, its interpretation necessitates further investigation. As was observed in the results from swimming pools in Italy (Fantuzzi et al. 2018), in this study, cocaine was detected in every season; furthermore, it was found in every sample in the pre-season. This finding indicates that cocaine consumption is also widespread among the local population, as Thomas et al. (2012) and Mackul’ak et al. (2016) have also revealed. However, the absolute peaks were observed in the summer at spas frequented by tourists (Table S3).

**Fig. 3:**
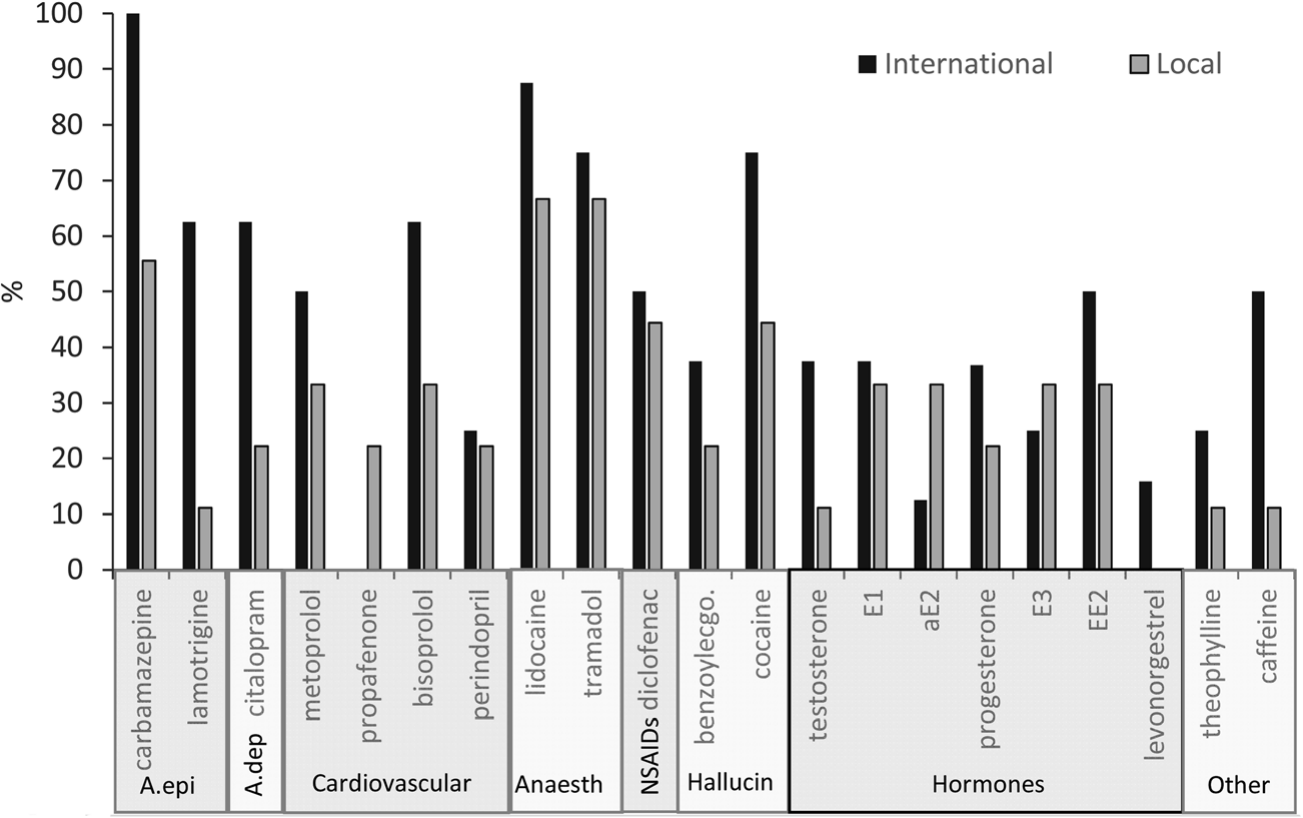
Geographical-based frequency variation of the detected PhACs (%) of all seasons; A.epi: antiepilepticum; A.dep: antidepressants; Cardiovascular: cardiovascular drugs; Anaesth: anaesthetics and analgesics; NSAIDs: nonsteroidal anti-inflammatory drugs; Hallucin: stimulants, hallucinogens and their metabolites

Regarding geographical variation, research has shown that, as compared to spas mainly visited by the locals, nearly all PhACs occur more frequently at international thermal spas within the city centre, and that the average concentrations of the detected PhACs are higher, especially in the cases of cocaine and certain hormones. The exceptions are the two forms of oestrogen and the cardiovascular drug propafenone, which occur more frequently at spas located outside of the city (Fig. 3).

### Diurnal analysis

**Fig. 4:**
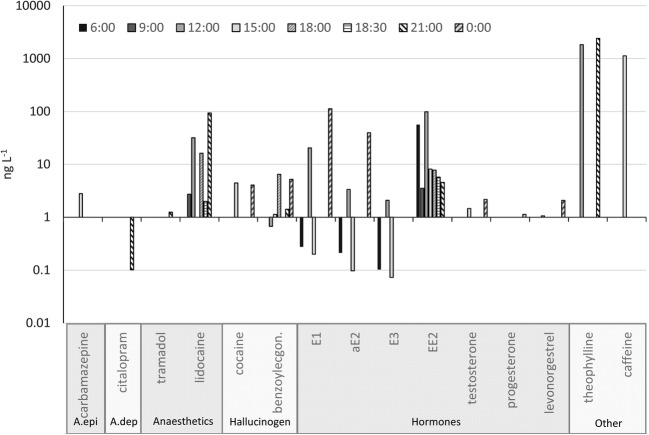
Diurnal fluctuation of PhAC concentrations in the DTWW outflow of Spa A on 26 July 2018; A.epi: antiepilepticum; A.dep: antidepressants; Anaesthetics: anaesthetics and analgesics; Hallucinogen: stimulants, hallucinogens and their metabolites

Eight water samples were collected for Spa A diurnal analysis, and only 15 of the 111 possible PhACs were detected (Fig. 4). It should be noted that nearly half of the identified PhACs were hormones, and that the occurrence (and non-occurrence) of many other agents were atypical.

EE2 was identified at high concentrations (average: 23.1 ng L^−1^), and with a wide range (4-98 ng L^−1^, in seven of the eight samples (coefficient of variation, CV = 140%). The oestrogens were found to dynamically fluctuate, exhibiting no apparent patterns. Different types of hormones (testosterone, progesterone, levonorgestrel) were only occasionally measured at low concentrations.

Of the anaesthetics, lidocaine was dominant in terms of frequency and concentration. Nevertheless, the concentration of this substance relevantly fluctuated (CV = 130%), and it was absent in three samples, indicating fast water replacement. Additionally, the steadily high concentration of EE2 indicates persistent contamination throughout the entire day. It is also noteworthy that the typically frequently detected carbamazepine (Aus der Beek et al. 2016; Heberer 2002) was detected only once (in the afternoon sample), and that the concentration of diclofenac was found to be zero. Although they were detected in only three samples, alkaloids were found to have the highest concentration, i.e. >1 μg L^−1^ in each case; this also indicates fast water replacement and no accumulation. Additionally, although cocaine was detected in only two of the eight samples, its metabolite (benzoylecgonine) was present in five samples.

Regarding daily distribution, an absolute peak was found in the number of PhACs measured in sample of 15:00 at Spa A, when nine PhACs were found. The next highest peaks occurred in the noon and midnight samples. The 9:00 sample, and the sample containing the murky water observed at 18:30 (according to general water chemistry, this sample was due to pool rinsing), were found to have the fewest PhACs, even though both samples also contained lidocaine and EE2.

**Fig. 5:**
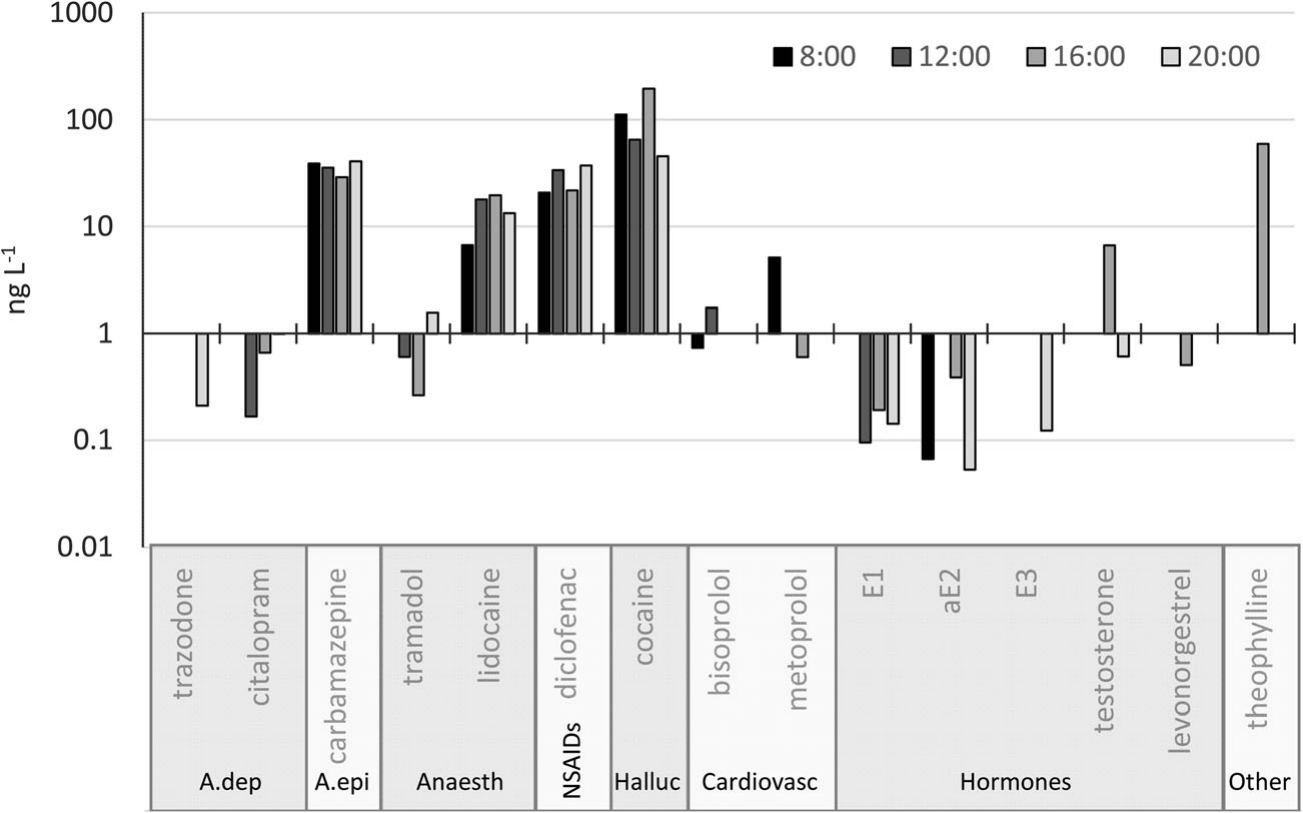
Diurnal fluctuation of various PhAC concentrations in the thermal water discharged from Spa B on 12 August 2018; A.dep: antidepressants; A.epi: antiepilepticum; Anaesth: anaesthetics and analgesics; NSAIDs: nonsteroidal anti-inflammatory drugs; Halluc: stimulants, hallucinogens and their metabolites; Cardiovasc: cardiovascular drugs

Regarding the diurnal analysis for Spa B, which has international visitors, of the 15 PhACs that were found, only one-third of them were hormones (Fig. 5). Some of the detected hormones were also found at Spa A (e.g. E1, E2, estriol); however, the summer diurnal analysis for Spa B did not yield EE2; furthermore, it was only detected once at Spa B (26 July 2018). Although several types of hormones were found, their concentrations were not high; specifically, with the exception of the testosterone measured in one sample, all hormones remained below 1 ng L^−1^. Additionally, only the concentration of E1 was stable, as the concentrations of the other hormones fluctuated throughout the day; specifically, their occurrence was inconsistent.

The concentrations of the anti-inflammatory drug diclofenac and anaesthetic lidocaine were found to be high (typically >10 ng L^−1^), moreover, the time of day did not relevantly affect the concentrations of these compounds (CV = 40% and 30%, respectively). Because there was no accumulation, the persistent presence of these compounds indicates continuous and largely invariable levels of contamination. The concentration of the antiepileptic drug carbamazepine (CV = 14%) was also found to be high and very stable; specifically, the concentration was considerably higher (36.1 ng L^−1^) than the swimming-pool-water average (1.1 ng L^−1^) measured by Fantuzzi et al. (2018). Although Fantuzzi et al. (2018) detected carbamazepine metabolites at concentrations up to 62 ng L^−1^, their accumulation resulting from water recirculation should be taken into account. Thus, the persistently high concentration as a result of continuous contamination is rather relevant. Additionally, although it fluctuated (CV = 63%), the concentration of cocaine was found to be the highest; furthermore, the concentration remained high throughout the day. The concentration of cocaine measured at Spa B within a single day (46-194 ng L^−1^, average: 104.2 ng L^−1^) was found to be higher, by two orders of magnitude, than the corresponding swimming pool measurement by Fantuzzi et al. (2018) (average: 1.29 ng L^−1^), and the average of the data used for geographical analysis (4.8 and 1.3 ng L^−1^ for international and local spas, respectively). It should be noted that, although the cocaine metabolite benzoylecgonine was detected in several samples from Spa A under the condition of low cocaine occurrence, this metabolite was not detected at Spa B. This is unexpected, as benzoylecgonine is much more stable than cocaine and the concentration of the former is generally higher (McCall et al. 2016; Thiebault et al. 2019). The background of this finding has been unknown yet, presumably, some other sources of cocaine (other than human metabolism) might have been present in the thermal water of Spa B.

Regarding daily fluctuation, the number and concentrations of PhACs were found to reach their peak in the early afternoon. Weng et al. (2014) and Teo et al. (2016) reported that caffeine could be a good indicator of urination and other types of excrement in swimming pools because it was consistently present at a high concentration, which was related to the number of visitors. However, this theory is not fully supported by the findings of this study; although one sample was found to have a high concentration of caffeine, it was absent in four of the samples (note: eight total samples). This supports the view that the measurements from swimming pools with strongly chlorinated water and water recirculation systems can only be indirectly compared to thermal spa systems.

Analysing all of the samples (off-season, pre-season, main season and diurnal monitoring) from the two international spas, which are similar in size and target the same type of visitors, revealed that the frequency and concentration of carbamazepine are constantly low at Spa A, unlike those at Spa B. However, as compared to Spa B, the frequent occurrence of hormones and constant presence and high concentration of EE2 at Spa A are relevant.

## Conclusions

The findings of this study reveal that significant amounts of PhACs enter thermal waters through the human body of visitors, and are then directly transported to surface waters. The measured concentrations indicate that thermal spas are not the main sources of contamination even though the emission of PhACs can still be relevant. The study itself, and the interpretation of the results, have some constraints. For example, the exact number, age and type (local inhabitants vs. tourists) of visitors who used the thermal pools during periods of sample collection are unknown. Furthermore, no previous studies, to which the results of this study can be compared, could be found. Nevertheless, our results can facilitate accurate assessment of the environmental pollution caused by DTWW, they also suggest the following:

- The concentrations and frequency of occurrence of PhACs contaminating the environment could be seasonal and dependent on the type of visitors.
- Many types of visitors use illicit drugs, as they were detected at international and local spas. However, although the concentrations of these PhACs increased at the time of an international music festival, there was no sudden change in the concentrations of other substances.
- As compared to the corresponding swimming pool measurements, PhACs remain in thermal pools for shorter periods of time, and at lower concentrations, because of the different filling and draining water treatment processes; furthermore, the types of PhACs can significantly change within a few hours in a thermal pool. Thus, the sampling time at thermal spas can be a critical determining factor. However, the concentrations of carbamazepine, diclofenac and cocaine, which are usually ubiquitous and very harmful to the environment, were negligible at one of the sampled spas, whereas the occurrence and concentrations of certain hormones were extremely high.
- Because the treatment and discharge technology and the type of visitors are not sufficient to justify such significant differences, it is likely that different microbial compositions and activity levels are contributing factors.
- Further research is required to better support the development of environmental risk reduction procedures.

## Electronic supplementary material

ESM 1 (DOCX 109 kb)
