# Supplementary material for: Thermal baths as sources of pharmaceutical and illicit drug contamination
Source: Environ Sci Pollut Res Int. 2019 Dec 2;27(1):399–410. doi: 10.1007/s11356-019-06633-6 (PMC6974506; doi:10.1007/s11356-019-06633-6)
Supplement: Supplementary file 1 — (DOCX 109 kb) [file 11356_2019_6633_MOESM1_ESM.docx]

Supplementary Information

**Thermal baths as sources of pharmaceutical and illicit drug contamination**

Gergely Jakab ^a, b, c, ^[[1]](#footnote-1)^^, Zoltán Szalai ^a, b, 1^, Gábor Michalkó ^a,g^, Marianna Ringer ^a^, Tibor Filep ^a^, Lili Szabó ^a, b^, Gábor Maász ^d^, Zsolt Pirger ^d^, Árpád Ferincz ^e^, Ádám Staszny ^e^, Péter Dobosy ^f^, Attila Csaba Kondor ^a, ^[[2]](#footnote-2)^*^

*^a^* *Geographical Institute, Research Centre for Astronomy and Earth Sciences, MTA Centre for Excellence, Budaörsi út 45., Budapest, H-1112 Hungary^b^Department of Environmental and Landscape Geography, Eötvös Loránd University, Pázmány Péter sétány 1/C., Budapest, H-1117, Hungary*

*^c^ Institute of Geography and Geoinformatics, University of Miskolc, Egyetemváros, Miskolc, H-3515, Hungary*

*^d^ MTA-Centre for Ecological Research, Balaton Limnological Institute, Klebelsberg Kuno u. 3., Tihany, H-8237, Hungary*

*^e^Department of Aquaculture, Szent István University, Páter K. u. 1., Gödöllő, H-2100, Hungary*

*^f^MTA-Centre for Ecological Research, Danube Research Institute, Karolina út 29., Budapest, H-1113, Hungary*

*^g^ Corvinus University of Budapest, Fővám tér 8., Budapest, H-1093 Hungary*

Table S1A. General chemical properties of the sampled outflow waters on 15.02.2018.

| **Spa** | A | C | D | E | F |
| --- | --- | --- | --- | --- | --- |
| **Date** | 15.02.2018 | | | | |
| **Temperature (°C)** | 30.7 | 30.0 | - | 30.8 | 37.5 |
| **pH** | 7.26 | 7.05 | 7.40 | 7.10 | 6.27 |
| **Specific electric conductivity (µS cm^-1^)** | 1500.0 | 1000.0 | 1150.0 | 1390.0 | 1480.0 |
| **Turbidity (NTU)** | <1 | <1 | <1 | <1 | <1 |
| **Hardness (°dH)** | 41.0 | 30.0 | 28.0 | 35.2 | 33.6 |
| **TOC (mg L^-1^)** | 1.62 | 0.49 | 0.58 | 0.39 | <0.1 |
| **TN (mg L^-1^)** | 0.91 | 0.96 | 0.54 | 0.14 | 0.57 |
| **Na^+^ (mg L^-1^)** | 131.69 | 72.33 | 92.47 | 100.52 | 141.62 |
| **NH^4+^ (mg L^-1^)** | 0.07 | 0.22 | 0.05 | <0.1 | 0.64 |
| **Mg^2+^ (mg L^-1^)** | 80.69 | 54.84 | 57.63 | 75.00 | 58.40 |
| **K^+^ (mg L^-1^)** | 17.81 | 9.59 | 12.40 | 13.15 | 15.74 |
| **Ca^2+^ (mg/L^-1^)** | 150.00 | 120.00 | 115.00 | 125.00 | 150.00 |
| **F^-^ (mg L^-1^)** | 2.28 | 1.46 | 2.32 | 3.10 | 0.11 |
| **Cl^-^ (mg L^-1^)** | 134.54 | 77.50 | 106.17 | 85.96 | 141.53 |
| **SO_4_^2-^ (mg L^-1^)** | 315.98 | 115.46 | 141.28 | 127.82 | 124.34 |
| **Br^-^(mg ^L-1^)** | 0.23 | 0.16 | 0.13 | 0.19 | 0.57 |
| **NO^3-^ (mg L^-1^)** | 3.14 | 3.45 | 2.54 | 1.79 | 1.57 |
| **NO^2-^ (mg L^-1^)** | <0.01 | <0.01 | 0.02 | <0.01 | <0.01 |
| **PO_4_^3-^ (mg L^-1^)** | <0.1 | <0.1 | 0.11 | <0.1 | <0.1 |
| **CO_3_^2-^ (mg L^-1^)** | <1 | <1 | <1 | <1 | <1 |
| **HCO^3-^ (mg L^-1^)** | 488.00 | 500.20 | 480.00 | 650.00 | 660.00 |
| **Cd (µg L^-1^)** | <0,01 | 0.01 | 0.03 | <0,01 | 0.08 |
| **Hg (µg L^-1^)** | 0.06 | 0.02 | 0.03 | <0,02 | 0.06 |
| **Pb (µg L^-1^)** | 0.06 | 0.05 | 0.02 | 0.04 | 0.11 |
| **Cr (µg L^-1^)** | 1.05 | 2.66 | 1.22 | 1.38 | 1.40 |
| **Ni (µg L^-1^)** | 1.05 | 2.22 | 1.10 | 1.15 | 1.27 |
| **Zn (µg L^-1^)** | 3.26 | 1.82 | 1.75 | 7.70 | 5.37 |
| **As (µg L^-1^)** | 10.30 | 2.33 | 1.17 | 1.69 | 4.41 |

Table S1B. General chemical properties of the sampled outflow waters on 10.06.2018.

| **Spa** | A | B | C | D | E | F |
| --- | --- | --- | --- | --- | --- | --- |
| **Date** | 10.06.2018 | | | | | |
| **Temperature (°C)** | 33.4 | 35.4 | 28.6 | 33.7 | 39.3 | 39.2 |
| **pH** | 7.16 | 7.01 | 7.02 | 7.14 | 6.84 | 6.82 |
| **Specific electric conductivity (µS cm^-1^)** | 1380.0 | 1580.0 | 810.0 | 1080.0 | 1410.0 | 1540.0 |
| **Turbidity (NTU)** | 1.0 | <1 | <1 | <1 | <1 | <1 |
| **Hardness (°dH)** | 30.5 | 48.5 | 20.6 | 39.0 | 25.2 | 45.2 |
| **TOC (mg L^-1^)** | 1.78 | 2.07 | 1.53 | 3.80 | 1.98 | 2.08 |
| **TN (mg L^-1^)** | 1.32 | 6.54 | 1.58 | 3.16 | 1.41 | 2.74 |
| **Na^+^ (mg L^-1^)** | 115.22 | 152.73 | 65.22 | 78.37 | 93.69 | 143.26 |
| **NH^4+^ (mg L^-1^)** | <0.1 | <0.1 | 0.14 | <0.1 | <0.1 | 0.44 |
| **Mg^2+^ (mg L^-1^)** | 39.00 | 138.91 | 31.76 | 90.00 | 70.00 | 138.04 |
| **K^+^ (mg L^-1^)** | 26.87 | 28.47 | 9.72 | 23.93 | 25.03 | 24.77 |
| **Ca^2+^ (mg/L^-1^)** | 158.73 | 80.77 | 96.88 | 134.04 | 80.00 | 75.80 |
| **F^-^ (mg L^-1^)** | 4.98 | 1.35 | 2.12 | 1.77 | 7.65 | 6.00 |
| **Cl^-^ (mg L^-1^)** | 124.14 | 242.73 | 78.87 | 186.02 | 90.83 | 183.61 |
| **SO_4_^2-^ (mg L^-1^)** | 282.26 | 245.96 | 100.56 | 123.22 | 100.00 | 124.97 |
| **Br^-^(mg ^L-1^)** | <0.1 | <0.1 | <0.1 | <0.1 | <0.1 | <0.1 |
| **NO^3-^ (mg L^-1^)** | 9.99 | 27.25 | 6.05 | 12.73 | 9.82 | 14.31 |
| **NO^2-^ (mg L^-1^)** | <0.01 | 0.01 | <0.01 | <0.01 | <0.01 | <0.01 |
| **PO_4_^3-^ (mg L^-1^)** | 0.09 | 0.11 | 0.08 | 0.49 | 0.16 | 0.07 |
| **CO_3_^2-^ (mg L^-1^)** | <1 | <1 | <1 | <1 | <1 | <1 |
| **HCO^3-^ (mg L^-1^)** | 390.40 | 500.00 | 414.80 | 550.00 | 650.00 | 720.00 |
| **Cd (µg L^-1^)** | 0.01 | 0.04 | <0,01 | 0.02 | 0.02 | 0.06 |
| **Hg (µg L^-1^)** | 0.19 | 0.04 | 0.10 | 0.08 | 0.04 | 0.04 |
| **Pb (µg L^-1^)** | 0.05 | 0.19 | 0.02 | 0.05 | 0.05 | 0.06 |
| **Cr (µg L^-1^)** | 3.97 | 1.85 | 2.84 | 2.57 | 2.72 | 2.05 |
| **Ni (µg L^-1^)** | 3.39 | 3.11 | 2.30 | 2.12 | 2.84 | 2.15 |
| **Zn (µg L^-1^)** | 8.06 | 5.75 | 3.40 | 12.26 | 5.82 | 4.81 |
| **As (µg L^-1^)** | 17.41 | 3.92 | 4.62 | 6.85 | 2.35 | 2.25 |

Table S1C. General chemical properties of the sampled outflow waters on 26.07.2018.

| **Spa** | A | B | C | D | E | F |
| --- | --- | --- | --- | --- | --- | --- |
| **Date** | 26.07.2018 | | | | | |
| **Temperature (°C)** | - | 35.0 | 29.4 | 33.0 | 38.3 | 40.0 |
| **pH** | 7.38 | 6.84 | 7.32 | 7.26 | 6.75 | 6.86 |
| **Specific electric conductivity (µS cm^-1^)** | 1380.0 | 1750.0 | 830.0 | 810.0 | 1230.0 | 1400.0 |
| **Turbidity (NTU)** | 1.5 | 1.1 | 10.0 | 1.3 | <1 | <1 |
| **Hardness (°dH)** | 42.6 | 42.6 | 28.0 | 28.5 | 36.9 | 38.5 |
| **TOC (mg L^-1^)** | 2.40 | 3.43 | 2.77 | 1.52 | 2.46 | 1.75 |
| **TN (mg L^-1^)** | 1.72 | 6.26 | 3.47 | 2.06 | 2.57 | 2.45 |
| **Na^+^ (mg L^-1^)** | 122.82 | 143.96 | 68.28 | 58.68 | 79.45 | 136.75 |
| **NH^4+^ (mg L^-1^)** | <0.1 | 0.20 | 0.64 | <0.1 | <0.1 | 0.55 |
| **Mg^2+^ (mg L^-1^)** | 90.70 | 72.26 | 61.45 | 63.83 | 77.29 | 75.02 |
| **K^+^ (mg L^-1^)** | 19.57 | 19.69 | 9.86 | 12.16 | 13.77 | 15.75 |
| **Ca^2+^ (mg/L^-1^)** | 120.00 | 172.06 | 93.16 | 103.84 | 161.28 | 160.33 |
| **F^-^ (mg L^-1^)** | 2.84 | 2.42 | 0.29 | 1.98 | 3.37 | 3.84 |
| **Cl^-^ (mg L^-1^)** | 127.30 | 228.06 | 185.93 | 89.73 | 79.90 | 163.17 |
| **SO_4_^2-^ (mg L^-1^)** | 299.53 | 236.79 | 58.35 | 92.00 | 129.53 | 124.38 |
| **Br^-^(mg ^L-1^)** | <0.1 | <0.1 | <0.1 | <0.1 | <0.1 | <0.1 |
| **NO^3-^ (mg L^-1^)** | 4.82 | 23.57 | 9.82 | 7.15 | 9.76 | 8.96 |
| **NO^2-^ (mg L^-1^)** | 0.01 | 0.04 | 0.01 | 0.02 | 0.01 | 0.02 |
| **PO_4_^3-^ (mg L^-1^)** | 0.15 | 0.16 | 0.45 | 0.45 | 0.22 | 0.16 |
| **CO_3_^2-^ (mg L^-1^)** | <1 | <1 | <1 | <1 | <1 | <1 |
| **HCO^3-^ (mg L^-1^)** | 512.40 | 475.80 | 350.00 | 450.00 | 880.00 | 928.00 |
| **Cd (µg L^-1^)** | 0.02 | 0.03 | 0.03 | 0.03 | 0.02 | 0.01 |
| **Hg (µg L^-1^)** | 0.10 | 0.06 | 0.10 | 0.18 | 0.04 | 0.25 |
| **Pb (µg L^-1^)** | 0.02 | 0.09 | 0.03 | 0.07 | 0.04 | 0.05 |
| **Cr (µg L^-1^)** | 0.79 | 2.70 | 1.05 | 1.12 | 0.74 | 1.54 |
| **Ni (µg L^-1^)** | 0.87 | 2.20 | 0.78 | 0.86 | 0.37 | 1.21 |
| **Zn (µg L^-1^)** | 5.58 | 3.90 | 6.16 | 3.99 | 4.57 | 5.99 |
| **As (µg L^-1^)** | 8.27 | 13.40 | 4.13 | 1.81 | 1.70 | 3.80 |

Table S1D. General chemical properties of the sampled outflow of Spa A on 26.07.2018.

| **Time (hh:mm)** | 06:00 | 09:00 | 12:00 | 15:00 | 18:00 | 18:30 | 21:00 | 24:00 |
| --- | --- | --- | --- | --- | --- | --- | --- | --- |
| **Temperature (°C)** | 39.4 | 30.8 | 32.6 | 35.0 | - | - | - | - |
| **pH** | 7.30 | 7.48 | 7.55 | 7.59 | 7.38 | 7.43 | 7.35 | 7.37 |
| **Specific electric conductivity (µS cm^-1^)** | 1240.0 | 1310.0 | 1270.0 | 1290.0 | 1380.0 | 1040.0 | 1340.0 | 1080.0 |
| **Turbidity (NTU)** | <1 | <1 | <1 | 1.2 | 1.5 | 9.1 | <1 | <1 |
| **Hardness (°dH)** | 35.0 | 34.6 | 35.5 | 40.0 | 42.6 | 22.4 | 37.0 | 31.0 |
| **TOC (mg L^-1^)** | 3.19 | 3.14 | 2.42 | 2.62 | 2.40 | 26.37 | 2.78 | 2.28 |
| **TN (mg L^-1^)** | 1.72 | 1.01 | 1.21 | 1.25 | 1.72 | 6.56 | 1.09 | 2.07 |
| **Na^+^ (mg L^-1^)** | 109.67 | 113.11 | 109.37 | 110.07 | 122.82 | 47.31 | 115.31 | 95.36 |
| **NH^4+^ (mg L^-1^)** | <0.1 | <0.1 | <0.1 | <0.1 | <0.1 | 0.05 | <0.1 | <0.1 |
| **Mg^2+^ (mg L^-1^)** | 70.00 | 81.88 | 78.62 | 77.48 | 90.70 | 47.30 | 88.36 | 66.00 |
| **K^+^ (mg L^-1^)** | 17.08 | 17.47 | 16.79 | 16.77 | 19.57 | 7.53 | 18.85 | 14.37 |
| **Ca^2+^ (mg/L^-1^)** | 120.00 | 154.80 | 120.00 | 130.00 | 120.00 | 70.06 | 115.00 | 105.00 |
| **F^-^ (mg L^-1^)** | 2.33 | 2.52 | 2.53 | 2.53 | 2.84 | 0.10 | 2.57 | 2.25 |
| **Cl^-^ (mg L^-1^)** | 117.27 | 121.85 | 122.40 | 123.15 | 127.30 | 53.54 | 121.16 | 117.84 |
| **SO_4_^2-^ (mg L^-1^)** | 239.33 | 276.74 | 258.56 | 260.55 | 299.53 | 88.65 | 289.13 | 190.59 |
| **Br^-^(mg ^L-1^)** | <0.1 | <0.1 | <0.1 | <0.1 | <0.1 | <0.1 | <0.1 | <0.1 |
| **NO^3-^ (mg L^-1^)** | 6.14 | 5.35 | 5.86 | 5.76 | 4.82 | 4.46 | 5.00 | 7.74 |
| **NO^2-^ (mg L^-1^)** | <0.01 | <0.01 | <0.01 | <0.01 | 0.01 | 0.02 | 0.01 | <0.01 |
| **PO_4_^3-^ (mg L^-1^)** | 0.18 | 0.17 | 0.19 | 0.19 | 0.15 | 0.32 | 0.14 | 0.40 |
| **CO_3_^2-^ (mg L^-1^)** | <1 | <1 | <1 | <1 | <1 | <1 | <1 | <1 |
| **HCO^3-^ (mg L^-1^)** | 463.60 | 524.60 | 488.00 | 475.80 | 512.40 | 402.60 | 500.20 | 402.60 |
| **Cd (µg L^-1^)** | 0.02 | 0.02 | 0.04 | 0.01 | 0.02 | 0.04 | 0.01 | 0.02 |
| **Hg (µg L^-1^)** | 0.10 | 0.11 | 0.09 | 0.13 | 0.10 | 0.17 | 0.11 | 0.14 |
| **Pb (µg L^-1^)** | 0.11 | 0.02 | 0.03 | 0.05 | 0.02 | 0.05 | 0.02 | 0.10 |
| **Cr (µg L^-1^)** | 0.65 | 0.53 | 0.99 | 1.18 | 0.79 | 1.40 | 0.65 | 1.31 |
| **Ni (µg L^-1^)** | 0.75 | 0.65 | 1.16 | 0.79 | 0.87 | 1.87 | 0.83 | 1.59 |
| **Zn (µg L^-1^)** | 5.86 | 3.77 | 4.84 | 5.15 | 5.58 | 2.59 | 4.54 | 4.42 |
| **As (µg L^-1^)** | 10.04 | 11.93 | 1.89 | 10.75 | 8.27 | 2.99 | 11.11 | 13.80 |

Table S1E. General chemical properties of the sampled outflow of Spa B on 12.08.2018.

| **Time (hh:mm)** | 08:00 | 12:00 | 16:00 | 20:00 |
| --- | --- | --- | --- | --- |
| **Temperature (°C)** | 31.3 | 33.2 | 34.0 | 31.3 |
| **pH** | 6.37 | 6.26 | 6.21 | 6.26 |
| **Specific electric conductivity (µS cm^-1^)** | 1580.0 | 1740.0 | 1660.0 | 1610.0 |
| **Turbidity (NTU)** | <1 | 1.0 | 1.6 | 1.2 |
| **Hardness (°dH)** | 45.2 | 42.0 | 40.0 | 36.0 |
| **TOC (mg L^-1^)** | 1.94 | 1.98 | 3.43 | 2.17 |
| **TN (mg L^-1^)** | 6.61 | 6.75 | 6.91 | 6.93 |
| **Na^+^ (mg L^-1^)** | 140.49 | 150.89 | 143.19 | 133.48 |
| **NH^4+^ (mg L^-1^)** | 0.30 | 0.22 | 0.08 | 0.18 |
| **Mg^2+^ (mg L^-1^)** | 90.00 | 90.00 | 80.00 | 80.00 |
| **K^+^ (mg L^-1^)** | 24.65 | 23.46 | 22.66 | 20.88 |
| **Ca^2+^ (mg/L^-1^)** | 175.00 | 165.00 | 150.00 | 120.00 |
| **F^-^ (mg L^-1^)** | 3.19 | 0.21 | 0.21 | 0.21 |
| **Cl^-^ (mg L^-1^)** | 219.22 | 229.64 | 231.76 | 207.84 |
| **SO_4_^2-^ (mg L^-1^)** | 225.83 | 237.00 | 226.37 | 220.42 |
| **Br^-^(mg ^L-1^)** | <0.1 | <0.1 | <0.1 | <0.1 |
| **NO^3-^ (mg L^-1^)** | 26.84 | 25.55 | 24.73 | 26.97 |
| **NO^2-^ (mg L^-1^)** | 0.03 | 0.03 | 0.07 | 0.04 |
| **PO_4_^3-^ (mg L^-1^)** | 0.10 | <0.1 | <0.1 | <0.1 |
| **CO_3_^2-^ (mg L^-1^)** | <1 | <1 | <1 | <1 |
| **HCO^3-^ (mg L^-1^)** | 650 | 600 | 520 | 450 |
| **Cd (µg L^-1^)** | 0.04 | 0.04 | 0.05 | 0.04 |
| **Hg (µg L^-1^)** | 0.07 | 0.08 | 0.11 | 0.07 |
| **Pb (µg L^-1^)** | 0.35 | 0.21 | 0.25 | 0.25 |
| **Cr (µg L^-1^)** | 0.97 | 1.54 | 2.94 | 1.95 |
| **Ni (µg L^-1^)** | 2.83 | 3.35 | 4.60 | 3.97 |
| **Zn (µg L^-1^)** | 7.34 | 6.05 | 8.07 | 6.33 |
| **As (µg L^-1^)** | 2.46 | 2.77 | 2.67 | 2.45 |

**Table S2** Validation parameters of the investigated PhACs

| **Chemical class** | **PhACs' name** | **Ionization mode** | **Precursor ion [m/z]** | **Quantifier ion [m/z]** | **CE [V]** | **Product ion [m/z]** | **CE [V]** | **Product ion [m/z]** | **CE [V]** | **Product ion [m/z]** | **CE [V]** | **Product ion [m/z]** | **CE [V]** | **t_R_ [min]** | **LOD [ng/L]** | **LOQ [ng/L]** | **Linearity range [ng/L]** | **R^2^** | **Measured Eluate** |
| --- | --- | --- | --- | --- | --- | --- | --- | --- | --- | --- | --- | --- | --- | --- | --- | --- | --- | --- | --- |
| Alkaloids | atropine | positive | 290.00 | 102.95 | 45 | 123.95 | 25 | 92.95 | 30 | 260.00 | 20 | 76.95 | 40 | 5.100 | 0.01 | 0.05 | 0.05-100 | 0.9836 | 2 |
|  | caffeine | positive | 194.95 | 137.95 | 20 | 109.95 | 20 | 92.95 | 20 | 82.95 | 25 |  |  | 2.370 | 5.00 | 10.00 | 10-20000 | 0.9862 | 1 |
|  | drotaverin | positive | 398.15 | 354.18 | 30 | 370.22 | 25 | 326.15 | 35 | 282.12 | 40 | 340.15 | 35 | 3.420 | 0.07 | 0.10 | 0.1-200 | 0.9959 | 2 |
|  | papaverin | positive | 340.00 | 202.00 | 25 | 324.15 | 30 | 296.00 | 30 | 170.95 | 35 | 280.00 | 45 | 3.150 | 0.03 | 0.10 | 0.1-200 | 0.9805 | 2 |
|  | scopolamine | positive | 304.15 | 137.95 | 25 | 155.95 | 15 | 120.95 | 20 | 109.95 | 25 | 102.95 | 35 | 3.280 | 0.01 | 0.05 | 0.05-100 | 0.9937 | 2 |
|  | theophyllin | positive | 180.95 | 123.95 | 15 | 95.95 | 20 | 68.95 | 25 | 41.95 | 25 |  |  | 2.640 | 5.00 | 10.00 | 10-20000 | 0.9927 | 1 |
| Antiepi-leptics | carbamazepine | positive | 237.10 | 194.10 | 20 | 179.05 | 30 | 165.05 | 35 | 152.10 | 35 | 220.10 | 15 | 3.030 | 0.02 | 0.10 | 0.05-100 | 0.9952 | 1 |
|  | lacosamide | positive | 251.12 | 108.10 | 10 | 91.10 | 10 | 116.08 | 15 | 219.12 | 6 | 74.10 | 25 | 2.700 | 0.10 | 0.50 | 0.5-1000 | 0.9488 | 1 |
|  | lamotrigine | positive | 256.02 | 211.00 | 25 | 144.95 | 35 | 109.00 | 45 | 159.00 | 25 | 187.00 | 25 | 4.270 | 1.15 | 5.00 | 5-10000 | 0.9792 | 2 |
|  | levetiracetam | positive | 171.15 | 126.05 | 15 | 154.10 | 6 | 69.05 | 25 |  |  |  |  | 2.790 | 80.00 | 200.00 | 200-400000 | 0.9786 | 2 |
| Antipsychotics/Antidepressants | amitriptillin | positive | 278.00 | 233.00 | 15 | 190.95 | 20 | 116.95 | 20 | 104.95 | 20 | 90.95 | 20 | 3.470 | 0.03 | 0.10 | 0.1-200 | 0.9850 | 2 |
|  | aripirazol | positive | 448.20 | 285.12 | 25 | 218.15 | 25 | 176.05 | 30 | 146.05 | 45 | 98.10 | 35 | 4.010 | 0.05 | 0.10 | 0.1-200 | 0.9988 | 2 |
|  | bupropion | positive | 240.00 | 183.95 | 10 | 165.95 | 20 | 138.95 | 25 | 130.95 | 25 | 102.95 | 35 | 1.760 | 0.15 | 0.50 | 0.5-1000 | 0.9418 | 2 |
|  | chlorpromazine | positive | 319.15 | 85.95 | 20 | 246.00 | 20 | 238.00 | 20 | 57.95 | 20 |  |  | 3.600 | 0.10 | 0.50 | 0.5-1000 | 0.9949 | 2 |
|  | citalopram | positive | 325.15 | 108.95 | 25 | 280.00 | 15 | 262.00 | 20 | 234.00 | 25 | 165.95 | 25 | 4.060 | 0.03 | 0.10 | 0.1-200 | 0.9981 | 2 |
|  | clozapine | positive | 327.15 | 270.00 | 25 | 296.00 | 25 | 227.00 | 25 | 191.95 | 40 | 83.95 | 20 | 4.260 | 0.03 | 0.10 | 0.1-200 | 0.9914 | 2 |
|  | cyproheptadine | positive | 288.00 | 190.95 | 30 | 95.95 | 25 | 195.95 | 20 | 215.00 | 40 | 109.95 | 20 | 3.560 | 0.15 | 0.50 | 0.5-1000 | 0.9927 | 2 |
|  | droperidol | positive | 380.15 | 164.95 | 25 | 193.95 | 15 | 122.95 | 45 | 94.95 | 60 |  |  | 3.710 | 0.01 | 0.10 | 0.1-200 | 0.9785 | 1 |
|  | duloxetine | positive | 298.00 | 267.00 | 15 | 239.00 | 25 | 122.95 | 20 | 182.95 | 20 | 156.95 | 25 | 3.910 | 1.00 | 5.00 | 5-10000 | 0.9095 | 2 |
|  | fluoxetine | positive | 310.15 | 147.95 | 5 | 259.00 | 15 | 251.00 | 20 | 290.00 | 10 | 43.95 | 5 | 3.530 | 0.01 | 0.50 | 0.5-1000 | 0.9882 | 2 |
|  | haloperidol | positive | 376.15 | 164.95 | 20 | 206.00 | 25 | 193.95 | 20 | 358.15 | 20 | 122.95 | 40 | 4.030 | 0.01 | 0.10 | 0.1-200 | 0.9872 | 2 |
|  | mCPP | positive | 196.95 | 154.05 | 20 | 119.08 | 20 | 118.10 | 30 | 111.02 | 30 | 104.05 | 30 | 3.580 | 0.50 | 5.00 | 5-10000 | 0.9839 | 2 |
|  | metoclopramide | positive | 300.15 | 227.00 | 20 | 183.95 | 30 | 140.95 | 45 | 112.95 | 55 | 89.95 | 45 | 5.620 | 0.01 | 0.20 | 0.2-400 | 0.9822 | 2 |
|  | mianserin | positive | 265.00 | 208.00 | 20 | 90.95 | 40 | 192.95 | 35 | 57.95 | 40 | 117.95 | 40 | 2.740 | 0.02 | 0.10 | 0.1-200 | 0.9849 | 1 |
|  | mirtazapine | positive | 266.00 | 194.95 | 25 | 223.00 | 20 | 209.00 | 20 | 235.00 | 20 | 71.95 | 20 | 3.380 | 0.15 | 0.10 | 0.1-200 | 0.9635 | 1 |
|  | olanzapine | positive | 313.15 | 256.00 | 20 | 282.00 | 20 | 213.00 | 30 | 197.95 | 40 | 83.90 | 20 | 4.610 | 1.00 | 5.00 | 5-10000 | 0.9542 | 2 |
|  | paliperidone | positive | 427.20 | 207.00 | 25 | 109.95 | 40 | 178.95 | 40 | 164.95 | 40 | 81.95 | 40 | 4.380 | 0.03 | 0.10 | 0.1-200 | 0.9853 | 2 |
|  | paroxetine | positive | 330.15 | 191.95 | 20 | 150.95 | 20 | 122.95 | 25 | 108.95 | 30 | 69.95 | 25 | 4.040 | 0.50 | 5.00 | 5-10000 | 0.9846 | 2 |
|  | procyclidine | positive | 288.00 | 83.95 | 20 | 94.95 | 25 | 90.95 | 40 | 270.00 | 15 | 55.95 | 40 | 3.690 | 0.02 | 0.20 | 0.2-400 | 0.9535 | 2 |
|  | quetiapine | positive | 384.15 | 253.00 | 20 | 221.00 | 35 | 279.00 | 25 | 210.00 | 35 | 157.95 | 20 | 3.700 | 0.05 | 0.10 | 0.1-200 | 0.9813 | 2 |
|  | risperidone | positive | 411.20 | 190.95 | 30 | 162.95 | 45 | 109.95 | 50 | 81.95 | 50 | 68.95 | 50 | 4.490 | 0.05 | 0.10 | 0.1-200 | 0.9203 | 2 |
|  | sertraline | positive | 306.15 | 158.95 | 20 | 196.95 | 15 | 275.00 | 10 | 128.95 | 20 | 90.95 | 20 | 3.390 | 3.00 | 5.00 | 5-10000 | 0.9827 | 2 |
|  | tiapride | positive | 329.15 | 256.00 | 20 | 213.00 | 30 | 133.95 | 45 | 176.95 | 35 | 241.00 | 30 | 5.040 | 0.01 | 0.10 | 0.1-200 | 0.9786 | 2 |
|  | trazodone | positive | 372.15 | 175.95 | 20 | 147.95 | 30 | 132.95 | 35 | 119.95 | 50 | 77.95 | 50 | 3.120 | 0.01 | 0.05 | 0.05-100 | 0.9794 | 2 |
| Anxiolytics | 7-aminoflunitrazepam | positive | 284.00 | 134.95 | 25 | 256.00 | 20 | 236.00 | 25 | 227.00 | 25 | 264.00 | 20 | 3.740 | 0.30 | 0.10 | 0.1-200 | 0.9567 | 2 |
|  | alprazolam | positive | 309.15 | 281.00 | 25 | 205.00 | 40 | 274.00 | 25 | 241.00 | 25 | 164.95 | 30 | 3.660 | 0.01 | 0.10 | 0.1-200 | 0.9963 | 2 |
|  | buspirone | positive | 386.15 | 121.95 | 30 | 222.00 | 25 | 149.95 | 25 | 265.00 | 25 | 108.95 | 35 | 3.110 | 0.01 | 0.10 | 0.1-200 | 0.9732 | 2 |
|  | chlordiazepoxide | positive | 300.15 | 227.00 | 25 | 282.00 | 25 | 255.00 | 20 | 241.00 | 20 | 283.00 | 15 | 3.240 | 0.15 | 0.50 | 0.5-1000 | 0.9805 | 2 |
|  | cinolazepam | positive | 358.15 | 312.15 | 20 | 340.15 | 15 | 272.00 | 35 | 245.00 | 40 | 210.00 | 45 | 2.670 | 0.03 | 0.10 | 0.1-200 | 0.9887 | 1 |
|  | clonazepam | positive | 316.15 | 270.00 | 25 | 214.00 | 40 | 241.00 | 35 | 207.00 | 30 | 150.95 | 55 | 2.930 | 0.02 | 0.10 | 0.1-200 | 0.9791 | 1 |
|  | diclazepam | positive | 319.15 | 227.00 | 30 | 262.00 | 25 | 256.00 | 25 | 291.00 | 20 | 153.95 | 30 | 2.340 | 0.15 | 0.50 | 0.5-1000 | 0.9943 | 1 |
|  | flumazenil | positive | 304.15 | 258.00 | 15 | 161.95 | 35 | 229.00 | 25 | 217.00 | 25 |  |  | 2.730 | 0.01 | 0.10 | 0.1-200 | 0.9869 | 1 |
|  | meprobamate | positive | 219.00 | 158.12 | 10 | 97.15 | 15 | 69.12 | 15 | 55.10 | 20 |  |  | 2.980 | 1.50 | 5.00 | 5-10000 | 0.9917 | 1 |
|  | midazolam | positive | 326.15 | 291.00 | 25 | 249.00 | 35 | 244.00 | 25 | 128.95 | 40 |  |  | 3.220 | 0.01 | 0.10 | 0.1-200 | 0.9969 | 2 |
|  | nitrazepam | positive | 282.00 | 236.00 | 25 | 207.00 | 35 | 179.95 | 35 | 151.95 | 55 | 189.95 | 40 | 2.890 | 0.03 | 0.10 | 0.1-200 | 0.9650 | 1 |
|  | nordiazepam | positive | 271.00 | 139.95 | 25 | 226.00 | 25 | 208.00 | 25 | 164.95 | 25 | 243.00 | 20 | 2.740 | 0.02 | 0.10 | 0.1-200 | 0.9701 | 1 |
|  | oxazepam | positive | 287.00 | 241.00 | 20 | 269.00 | 15 | 231.00 | 20 | 162.95 | 35 | 103.95 | 30 | 3.030 | 0.05 | 0.10 | 0.1-200 | 0.9918 | 1 |
|  | temazepam | positive | 301.15 | 255.00 | 20 | 283.00 | 15 | 228.00 | 20 | 192.95 | 35 | 176.95 | 35 | 2.330 | 0.05 | 0.10 | 0.1-200 | 0.9972 | 1 |
|  | zolpidem | positive | 308.15 | 235.00 | 35 | 263.00 | 25 | 221.00 | 35 | 91.95 | 50 | 248.00 | 35 | 3.420 | 0.01 | 0.01 | 0.1-200 | 0.9481 | 2 |
|  | zopiclone | positive | 389.15 | 245.00 | 20 | 217.00 | 30 | 345.15 | 8 | 138.95 | 40 |  |  | 3.450 | 0.05 | 0.10 | 0.1-200 | 0.9847 | 2 |
| Cardiovascular drugs | acenocoumarol | positive | 354.15 | 163.05 | 15 | 279.08 | 30 | 249.08 | 35 | 296.05 | 20 | 121.05 | 35 | 3.450 | 0.05 | 0.10 | 0.1-200 | 0.9873 | 1 |
|  | amiodarone | positive | 646.50 | 100.12 | 30 | 86.10 | 30 | 276.12 | 35 | 201.12 | 30 | 159.05 | 60 | 2.830 | 1.50 | 5.00 | 5-10000 | 0.9887 | 2 |
|  | amlodipine | positive | 409.20 | 238.05 | 10 | 294.10 | 10 | 220.05 | 25 | 206.05 | 25 | 170.05 | 30 | 4.300 | 0.30 | 5.00 | 5-10000 | 0.9982 | 2 |
|  | betaxolol | positive | 308.15 | 115.95 | 20 | 97.95 | 20 | 176.95 | 20 | 160.95 | 20 | 158.95 | 20 | 3.840 | 0.05 | 0.50 | 0.5-1000 | 0.9946 | 2 |
|  | bisoprolol | positive | 326.15 | 115.95 | 15 | 73.95 | 25 | 97.95 | 25 | 146.95 | 20 |  |  | 3.890 | 0.01 | 0.50 | 0.5-1000 | 0.9919 | 2 |
|  | carvedilol | positive | 407.20 | 224.00 | 20 | 222.00 | 20 | 283.00 | 20 | 179.95 | 20 | 99.95 | 25 | 4.850 | 0.20 | 0.10 | 0.1-200 | 0.9844 | 2 |
|  | cloranolol | positive | 292.00 | 236.00 | 15 | 218.00 | 20 | 201.00 | 20 | 174.95 | 25 | 144.95 | 40 | 3.380 | 0.03 | 0.10 | 0.1-200 | 0.9896 | 2 |
|  | esmolol | positive | 296.00 | 144.95 | 25 | 219.00 | 20 | 254.00 | 15 | 115.95 | 20 | 97.95 | 20 | 3.770 | 0.05 | 0.10 | 0.1-200 | 0.9985 | 2 |
|  | labetalol | positive | 329.20 | 162.05 | 25 | 207.10 | 25 | 91.05 | 30 | 294.15 | 20 |  |  | 3.650 | 0.02 | 0.10 | 0.1-200 | 0.9936 | 2 |
|  | losartan | positive | 423.20 | 207.00 | 20 | 179.95 | 35 | 235.00 | 20 | 377.15 | 15 | 405.20 | 10 | 5.380 | 0.02 | 0.10 | 0.1-200 | 0.9941 | 2 |
|  | metoprolol | positive | 268.00 | 115.95 | 20 | 158.95 | 20 | 190.95 | 15 | 97.95 | 20 | 132.95 | 25 | 3.790 | 0.02 | 0.10 | 0.1-200 | 0.9768 | 2 |
|  | nebivolol | positive | 406.20 | 150.95 | 30 | 176.95 | 25 | 388.15 | 20 | 122.95 | 40 | 102.95 | 50 | 4.080 | 1.00 | 5.00 | 5-10000 | 0.9952 | 2 |
|  | nifedipine | positive | 347.10 | 284.10 | 20 | 268.10 | 15 | 208.10 | 30 | 253.10 | 30 |  | 30 | 4.000 | 0.02 | 0.10 | 0.1-200 | 0.9955 | 2 |
|  | perindopril | positive | 369.15 | 171.95 | 20 | 295.00 | 15 | 169.95 | 20 | 97.95 | 30 | 71.95 | 25 | 4.040 | 0.02 | 0.10 | 0.1-200 | 0.9966 | 2 |
|  | pindolol | positive | 249.00 | 115.95 | 15 | 145.95 | 20 | 133.95 | 25 | 171.95 | 15 | 97.90 | 20 | 4.610 | 0.05 | 0.20 | 0.2-400 | 0.9426 | 2 |
|  | practolol | positive | 267.00 | 189.95 | 15 | 225.00 | 15 | 177.95 | 20 | 163.95 | 20 | 147.95 | 20 | 5.110 | 0.05 | 0.50 | 0.5-1000 | 0.9426 | 2 |
|  | prajmaline | positive | 369.15 | 157.95 | 40 | 327.15 | 30 | 143.95 | 45 | 130.95 | 45 | 121.95 | 35 | 5.350 | 5.00 | 20.00 | 20-40000 | 0.9801 | 2 |
|  | propafenone | positive | 342.15 | 115.95 | 20 | 265.00 | 20 | 324.15 | 15 | 97.95 | 20 | 71.95 | 25 | 4.010 | 0.05 | 0.50 | 0.5-1000 | 0.9944 | 2 |
|  | propranolol | positive | 260.00 | 115.95 | 15 | 182.95 | 15 | 156.95 | 20 | 154.95 | 25 | 97.95 | 15 | 3.830 | 0.01 | 0.10 | 0.1-200 | 0.9959 | 1 |
|  | trimetazidine | positive | 267.00 | 180.95 | 15 | 165.95 | 25 | 150.95 | 35 | 135.95 | 30 | 90.95 | 35 | 4.360 | 1.00 | 20.00 | 20-40000 | 0.9866 | 2 |
|  | verapamil | positive | 455.20 | 164.95 | 25 | 260.00 | 30 | 176.95 | 35 | 303.15 | 25 | 149.95 | 40 | 3.600 | 0.01 | 0.05 | 0.05-100 | 0.9923 | 2 |
|  | warfarin | positive | 309.15 | 162.95 | 15 | 251.00 | 20 | 291.00 | 10 | 146.95 | 15 | 120.95 | 40 | 2.790 | 0.03 | 0.10 | 0.1-200 | 0.9606 | 1 |
| Dissociative anesthetics/psychedelic drugs |  |  |  |  |  |  |  |  |  |  |  |  |  |  |  |  |  |  |  |
|  | ketamin | positive | 238.00 | 124.95 | 30 | 220.00 | 15 | 207.00 | 15 | 178.95 | 15 | 162.95 | 20 | 2.160 | 0.20 | 0.50 | 0.5-1000 | 0.9942 | 2 |
|  |  |  |  |  |  |  |  |  |  |  |  |  |  |  |  |  |  |  |  |
|  | norketamin | positive | 224.00 | 124.95 | 20 | 188.95 | 15 | 178.95 | 15 | 207.00 | 10 | 66.95 | 20 | 2.290 | 0.10 | 5.00 | 5-10000 | 0.9169 | 2 |
| Hormones/hormon derivatives | aE2 (dansyl) | positive | 506.20 | 170.95 | 35 | 114.95 | 70 | 155.95 | 55 | 425.20 | 30 | 440.20 | 25 | 2.140 | 0.01 | 0.05 | 0.05-100 | 0.9786 | 1^[[3]](#footnote-3)^* |
|  | bE2 (dansyl) | positive | 506.20 | 170.95 | 35 | 114.95 | 70 | 155.95 | 55 | 425.20 | 30 | 440.20 | 25 | 2.210 | 0.01 | 0.05 | 0.05-100 | 0.9813 | 1* |
|  | drospirenone | positive | 367.20 | 105.00 | 35 | 97.05 | 20 | 131.05 | 30 | 239.15 | 15 | 349.20 | 15 | 2.050 | 0.25 | 1.00 | 1-2000 | 0.9793 | 1* |
|  | E1 (dansyl) | positive | 504.20 | 170.95 | 35 | 114.95 | 70 | 155.95 | 55 | 425.20 | 30 | 440.20 | 25 | 1.760 | 0.01 | 0.05 | 0.05-100 | 0.9858 | 1* |
|  | E3 (dansyl) | positive | 522.20 | 170.95 | 35 | 114.95 | 70 | 155.95 | 55 | 425.20 | 30 | 440.20 | 25 | 2.850 | 0.01 | 0.05 | 0.05-100 | 0.9884 | 1* |
|  | EE2 (dansyl) | positive | 530.20 | 170.95 | 35 | 114.95 | 70 | 155.95 | 55 | 425.20 | 30 | 440.20 | 25 | 2.090 | 0.01 | 0.05 | 0.05-100 | 0.9782 | 1* |
|  | levonorgestrel | positive | 313.20 | 109.05 | 25 | 90.05 | 40 | 245.20 | 15 | 277.20 | 15 | 295.20 | 15 | 1.720 | 0.50 | 1.00 | 1-2000 | 0.9904 | 1* |
|  | progesterone | positive | 315.20 | 109.05 | 20 | 97.05 | 20 | 215.00 | 20 | 279.20 | 15 | 297.20 | 15 | 1.520 | 0.05 | 0.50 | 0.5-1000 | 0.9578 | 1* |
|  | testosterone | positive | 289.20 | 109.05 | 20 | 97.05 | 20 | 253.20 | 15 | 271.20 | 15 |  |  | 1.840 | 0.15 | 0.50 | 0.5-1000 | 0.9754 | 1* |
| Local anesthetics | benzocaine | positive | 165.95 | 119.95 | 15 | 137.95 | 10 | 93.95 | 15 | 91.95 | 25 | 76.95 | 25 | 1.830 | 5.00 | 20.00 | 20-40000 | 0.9718 | 1 |
|  | bupivacaine | positive | 289.00 | 140.12 | 20 | 98.15 | 35 | 84.10 | 40 |  |  |  |  | 2.310 | 0.01 | 0.10 | 0.1-200 | 0.9966 | 2 |
|  | lidocaine | positive | 235.00 | 86.10 | 15 | 58.10 | 30 |  |  |  |  |  |  | 1.920 | 0.05 | 0.10 | 0.1-200 | 0.9948 | 2 |
|  | nitracaine | positive | 309.15 | 149.95 | 25 | 236.00 | 15 | 141.95 | 20 | 85.95 | 25 | 68.95 | 20 | 1.050 | 0.01 | 0.10 | 0.1-200 | 0.9565 | 2 |
|  | procain | positive | 237.30 | 100.15 | 20 | 120.05 | 35 | 164.05 | 20 |  |  |  |  |  |  |  |  |  |  |
|  | ropivacain | positive | 275.00 | 125.95 | 20 | 83.95 | 35 | 97.95 | 35 | 149.95 | 20 | 55.95 | 40 | 2.340 | 0.01 | 0.10 | 0.1-200 | 0.9976 | 2 |
|  | tetracaine | positive | 265.00 | 175.95 | 15 | 220.00 | 15 | 119.95 | 35 | 91.95 | 35 | 71.95 | 20 | 3.240 | 0.03 | 0.10 | 0.1-200 | 0.8162 | 2 |
| NSAIDs | diclofenac | positive | 296.00 | 215.08 | 20 | 250.00 | 10 | 278.00 | 10 | 151.05 | 55 |  |  | 2.710 | 0.10 | 0.50 | 0.5-1000 | 0.9579 | 1 |
|  | fenacetin | positive | 179.85 | 109.95 | 20 | 137.95 | 15 | 151.95 | 15 | 92.95 | 25 | 64.95 | 30 | 2.690 | 0.05 | 0.50 | 0.5-1000 | 0.9975 | 2 |
|  | metamizol | positive | 218.00 | 186.95 | 10 | 158.95 | 10 | 124.95 | 10 | 96.95 | 15 | 55.95 | 15 | 3.760 | 15.00 | 200.00 | 200-400000 | 0.9584 | 1 |
|  | naproxen | positive | 231.00 | 184.95 | 15 |  |  | 169.95 | 25 | 152.95 | 30 | 140.95 | 40 | 2.390 | 0.20 | 0.10 | 0.1-200 | 0.9854 | 1 |
|  | paracetamol | positive | 151.95 | 110.05 | 15 | 93.05 | 20 | 82.10 | 25 | 43.08 | 20 |  |  | 3.610 | 3.50 | 20.00 | 20-40000 | 0.9337 | 1 |
| Opiods/morphine derivatives | 6-monoacetylmorphine | positive | 328.15 | 164.95 | 40 | 268.00 | 20 | 211.00 | 25 | 192.95 | 25 | 271.00 | 20 | 4.350 | 0.03 | 0.50 | 0.5-1000 | 0.9858 | 2 |
|  | codeine | positive | 300.15 | 215.00 | 25 | 243.00 | 20 | 225.00 | 25 | 282.00 | 20 | 164.95 | 35 | 4.240 | 0.10 | 5.00 | 5-10000 | 0.9911 | 2 |
|  | embutramide | positive | 294.00 | 120.95 | 25 | 134.95 | 20 | 148.95 | 20 | 190.95 | 15 | 208.00 | 15 | 2.990 | 0.05 | 0.10 | 0.1-200 | 0.9938 | 2 |
|  | ethylmorphine | positive | 314.15 | 229.00 | 25 | 257.00 | 20 | 239.00 | 25 | 296.00 | 20 | 164.95 | 35 | 4.210 | 0.15 | 0.50 | 0.5-1000 | 0.9899 | 2 |
|  | fentanyl | positive | 337.15 | 187.95 | 20 | 104.95 | 35 | 78.95 | 55 | 216.00 | 20 |  |  | 2.960 | 0.05 | 0.10 | 0.1-200 | 0.9876 | 1 |
|  | methadone | positive | 310.15 | 265.00 | 15 | 104.95 | 25 | 223.00 | 20 | 219.00 | 20 | 158.95 | 20 | 3.790 | 0.01 | 0.02 | 0.02-40 | 0.9933 | 2 |
|  | morphine | positive | 286.00 | 201.00 | 25 | 164.95 | 35 | 211.00 | 25 | 152.95 | 40 | 156.95 | 35 | 5.130 | 2.00 | 5.00 | 5-10000 | 0.9729 | 2 |
|  | nalbuphine | positive | 358.15 | 340.15 | 20 | 254.00 | 30 | 211.00 | 30 | 200.00 | 30 | 184.95 | 35 | 3.450 | 0.25 | 0.50 | 0.5-1000 | 0.9845 | 2 |
|  | oxycodone | positive | 316.15 | 298.00 | 20 | 256.00 | 25 | 241.00 | 25 | 212.00 | 40 | 186.95 | 25 | 3.400 | 0.10 | 5.00 | 5-10000 | 0.9271 | 2 |
|  | pethidine | positive | 248.00 | 220.00 | 20 | 202.00 | 15 | 173.95 | 20 | 130.95 | 30 | 69.95 | 30 | 3.070 | 0.02 | 0.10 | 0.1-200 | 0.9927 | 2 |
|  | tramadol | positive | 264.00 | 57.95 | 10 | 246.00 | 10 |  |  |  |  |  |  | 3.120 | 0.15 | 0.10 | 0.1-200 | 0.9984 | 2 |
| Others |  |  |  |  |  |  |  |  |  |  |  |  |  |  |  |  |  |  |  |
|  | atracurium | positive | 358.15 | 206.00 | 20 | 188.95 | 25 | 150.95 | 25 | 327.15 | 20 | 106.95 | 45 | 4.110 | 0.03 | 0.10 | 0.1-200 | 0.9844 | 2 |
|  | ephedrine | positive | 165.95 | 147.95 | 10 | 132.95 | 20 | 116.95 | 20 | 114.95 | 25 | 90.95 | 25 | 4.020 | 20.00 | 80.00 | 80-160000 | 0.9887 | 2 |
| Stimulants/Hallucinogens and their metabolites |  |  |  |  |  |  |  |  |  |  |  |  |  |  |  |  |  |  |  |
|  |  |  |  |  |  |  |  |  |  |  |  |  |  |  |  |  |  |  |  |
|  | benzoyl ecgonine^[[4]](#footnote-4)^+ | positive | 290.00 | 167.95 | 15 | 272.00 | 15 | 149.95 | 20 | 118.95 | 25 | 104.95 | 25 | 5.990 | 0.01 | 0.10 | 0.1-200 | 0.9972 | 2 |
|  | cocaine | positive | 304.15 | 181.95 | 20 | 149.95 | 25 | 90.95 | 30 | 104.95 | 30 |  |  | 2.440 | 0.01 | 0.05 | 0.05-100 | 0.9825 | 2 |
|  | dibutylon (N,N-dimethylbutylone) | positive | 236.00 | 190.95 | 15 | 148.95 | 25 | 160.95 | 20 | 162.95 | 20 | 132.95 | 25 | 1.880 | 0.10 | 0.20 | 0.2-400 | 0.9841 | 2 |
|  | dipentylone (N,N-dimethylpentylone) | positive | 250.00 | 205.00 | 15 | 174.95 | 20 | 148.95 | 25 | 134.95 | 25 | 99.95 | 20 | 1.810 | 0.03 | 0.20 | 0.2-400 | 0.9815 | 2 |

+metabolite of cocaine

* after derivatization

Table S2. List, groups and limit of quantification (LOQ) values of the analysed pharmaceutically active compounds (PhACs)

|  |  |  |
| --- | --- | --- |
|  |  |  |
|  |  |  |
|  |  |  |
|  |  |  |
|  |  |  |
|  |  |  |
|  |  |  |
|  |  |  |
|  |  |  |
|  |  |  |
|  |  |  |
|  |  |  |
|  |  |  |
|  |  |  |
|  |  |  |
|  |  |  |
|  |  |  |
|  |  |  |
|  |  |  |
|  |  |  |
|  |  |  |
|  |  |  |
|  |  |  |
|  |  |  |
|  |  |  |
|  |  |  |
|  |  |  |
|  |  |  |
|  |  |  |
|  |  |  |
|  |  |  |
|  |  |  |
|  |  |  |
|  |  |  |
|  |  |  |
|  |  |  |
|  |  |  |
|  |  |  |
|  |  |  |
|  |  |  |
|  |  |  |
|  |  |  |
|  |  |  |
|  |  |  |
|  |  |  |
|  |  |  |
|  |  |  |
|  |  |  |
|  |  |  |
|  |  |  |
|  |  |  |
|  |  |  |
|  |  |  |
|  |  |  |
|  |  |  |
|  |  |  |
|  |  |  |
|  |  |  |
|  |  |  |
|  |  |  |
|  |  |  |
|  |  |  |
|  |  |  |
|  |  |  |
|  |  |  |
|  |  |  |
|  |  |  |
|  |  |  |
|  |  |  |
|  |  |  |
|  |  |  |
|  |  |  |
|  |  |  |
|  |  |  |
|  |  |  |
|  |  |  |
|  |  |  |
|  |  |  |
|  |  |  |
|  |  |  |
|  |  |  |
|  |  |  |
|  |  |  |
|  |  |  |
|  |  |  |
|  |  |  |
|  |  |  |
|  |  |  |
|  |  |  |
|  |  |  |
|  |  |  |
|  |  |  |
|  |  |  |
|  |  |  |
|  |  |  |
|  |  |  |
|  |  |  |
|  |  |  |
|  |  |  |
|  |  |  |
|  |  |  |
|  |  |  |
|  |  |  |
|  |  |  |
|  |  |  |
|  |  |  |
|  |  |  |
|  |  |  |
|  |  |  |
|  |  |  |
|  |  |  |
|  |  |  |

Table S3A Mesured data (15.02.2018; ng L^-1^)

| Spa | A | C | D | E | F |
| --- | --- | --- | --- | --- | --- |
| Date | 15.02.2018 | | | | |
| caffeine | 484.96 |  |  |  |  |
| lidocaine | 27.91 | 0.96 | 0.81 |  |  |
| carbamazepine | 1.94 | 19.25 | 7.37 | 0.17 |  |
| lamotrigine | 72.25 | 18.49 |  |  |  |
| tramadol | 0.5 | 2.28 | 0.22 |  |  |
| metoprolol | 3.32 | 8.42 | 1.44 |  |  |
| benzoyl-ecgonine | 0.8 |  |  |  |  |
| cocaine | 4.89 |  |  | 0.46 |  |
| betaxolol | <LOQ |  | <LOQ |  |  |
| citalopram | 1.55 |  |  |  |  |
| bisoprolol | 1.37 | 0.89 | <LOQ | <LOQ |  |
| tiapride |  | 0.25 |  |  |  |
| cinolazepam |  | 0.36 |  |  |  |
| perindopril | 0.89 | 0.47 |  |  |  |
| bE2 | 5.6 |  |  |  |  |
| EE2 | 20.505 |  |  |  |  |
| testosteron | 97.31 |  |  |  |  |
| progesteron | 6.88 |  |  |  |  |
| levonorgestrel | 8.19 |  |  |  |  |

Table S3B Mesured data (10.06.2018; ng L^-1^)

| Spa | A | B | C | D | E | F |
| --- | --- | --- | --- | --- | --- | --- |
| Date | 10.06.2018 | | | | | |
| paracetamol |  | 76.06 |  |  |  |  |
| caffeine | 2061.43 | 1210.47 |  |  |  | 1701.69 |
| norketamin |  | 10.37 |  |  |  |  |
| lidocaine | 38.78 | 15.82 | 6.47 |  | 7.69 | 73.38 |
| carbamazepine | 3.04 | 188.57 | 48.58 |  |  | 63.51 |
| ketamin |  | 57 |  |  |  |  |
| bupropion | 1.16 |  |  |  |  |  |
| lamotrigine | 37.35 | 96.59 | 75.03 |  |  | 24.69 |
| tramadol | 0.71 | 2.61 | 2.65 | 0.6 | 14.96 | 1.35 |
| trimetazidine |  |  |  |  |  | <LOQ |
| metoprolol | 0.64 | 1.8 |  |  | 5.75 | 9.54 |
| diclofenac |  | 10.17 | 1.61 |  | 9.12 | 9.39 |
| cocaine | 0.85 | 7.85 | 0.14 | 0.81 | 3.64 | 0.21 |
| betaxolol | 1.12 | <LOQ | <LOQ |  |  |  |
| alprazolam |  |  |  |  |  |  |
| citalopram | 2.8 | 1.9 | 2.88 |  | 3.26 | 1.42 |
| bisoprolol | 2.46 |  | 1.58 | 1.3 | 8.1 | 13.27 |
| papaverin |  |  |  |  |  | 1.36 |
| propafenone |  |  |  |  | <LOQ | 0.91 |
| cinolazepam |  |  | <LOQ |  |  |  |
| perindopril | <LOQ |  |  |  | 0.49 | 0.24 |
| verapamil | 0.56 |  |  |  |  |  |
| testosteron |  | 9.77 |  |  | 39.63 |  |
| progesteron |  | 1.42 | 1.68 |  | 10.24 |  |

Table S3C Mesured data (Spa A, 26.07.2018; ng L^-1^)

| Spa | A_06h | A_09h | A_12h | A_15h | A_1830h | A_18h | A_21h | A_24h |
| --- | --- | --- | --- | --- | --- | --- | --- | --- |
|  |  |  |  |  |  |  |  |  |
| Date | 26.07.2018 | | | | | | | |
| theophylline |  |  | 1826.15 |  |  |  | 2405.19 |  |
| caffeine |  |  |  | 1126.39 |  |  |  |  |
| lidocaine |  | 2.7095 | 31.761 |  | 1.983 | 16.2245 | 93.4915 |  |
| carbamazepine |  |  |  | 2.7805 |  |  |  |  |
| tramadol |  |  |  |  |  |  | 1.2475 |  |
| benzoyl-ecgonine |  |  | 0.6735 | 1.131 |  | 6.471 | 1.412 | 5.221 |
| cocaine |  |  |  | 4.45 |  |  |  | 4.073 |
| citalopram |  |  |  |  |  |  | 0.1015 |  |
| E1 | 0.2765 |  | 20.4685 | 0.2 |  | <LOQ |  | 112.589 |
| aE2 | 0.2125 |  | 3.362 | 0.097 |  |  | <LOQ | 39.478 |
| E3 | 0.103 | <LOQ | 2.094 | 0.073 | <LOQ | <LOQ | <LOQ |  |
| EE2 | 56.338 | 3.535 | 98.327 | 8.165 | 5.733 | 7.785 | 4.5445 |  |
| testosteron |  |  | <LOQ | 1.4585 |  |  |  | 2.1785 |
| progesteron |  |  | <LOQ | 0.752 |  |  |  | 1.133 |
| levonorgestrel |  |  | 1.0615 |  |  |  |  | 2.0745 |

Table S3D Measured data (26.07.2018; ng L^-1^)

| Spa | B | C | D | E | F |
| --- | --- | --- | --- | --- | --- |
| Date | 26.07.2018 | | | | |
| theophylline | 7184.16 | 5915.08 | 2463.55 |  |  |
| lidocaine | 19.862 | 3.7785 | 132.863 | 5.2465 | 119.073 |
| carbamazepine | 33.1195 | 11.0875 |  | 2.219 | 20.1935 |
| tramadol |  | 1.887 | 0.884 |  | 1.431 |
| benzoyl-ecgonine | 3.9925 |  | 5.76 | 0.892 |  |
| diclofenac | 57.5915 | 4.882 | 32.533 |  | 53.1765 |
| cocaine | 10.58 |  |  |  |  |
| alprazolam |  |  |  | 0.5385 |  |
| citalopram | 1.422 |  |  |  |  |
| bisoprolol | 3.0635 |  |  |  |  |
| propafenone | <LOQ |  |  | 1.547 |  |
| E1 | 3.5385 | 0.786 | 0.088 | 5.937 | 0.071 |
| aE2 |  |  | 0.056 | 0.2715 | 0.1965 |
| E3 | 0.508 |  | 0.0505 | 1.09 | <LOQ |
| EE2 | 4.212 | 0.6415 | 4.8525 | 6.195 | 2.9895 |
| testosteron |  | <LOQ |  |  |  |
| progesteron | 2.201 |  |  | 2.019 |  |
| levonorgestrel | <LOQ | <LOQ |  |  |  |
| drospirenone |  | 1.8425 |  |  |  |

Table S3E Measured data (Spa B; 12.08.2018; ng L^-1^)

| Spa | B_08h | B_12h | B_16h | B_20h |
| --- | --- | --- | --- | --- |
| Date | 12.08.2018 | | | |
| theophylline |  |  | 59.432 |  |
| caffeine | 889.58 | 1170.22 | 1495.77 | 1984.32 |
| norketamin |  |  |  |  |
| lidocaine | 6.692 | 17.924 | 19.64 | 13.3895 |
| carbamazepine | 38.8695 | 35.5625 | 29.084 | 40.862 |
| tramadol |  | 0.6075 | 0.264 | 1.5765 |
| metoprolol | 5.116 |  | 0.603 |  |
| diclofenac | 20.664 | 33.638 | 21.7515 | 37.4605 |
| cocaine | 112.215 | 65.229 | 194.023 | 45.527 |
| betaxolol |  |  |  | <LOQ |
| citalopram |  | 0.167 | 0.6605 | 0.998 |
| bisoprolol | 0.7355 | 1.729 |  |  |
| trazodone |  |  |  | 0.211 |
| E1 | <LOQ | 0.095 | 0.191 | 0.1425 |
| aE2 | 0.0665 |  | 0.3885 | 0.053 |
| E3 | <LOQ | <LOQ | <LOQ | 0.123 |
| testosteron | <LOQ | <LOQ | 6.623 | 0.61 |
| progesteron |  |  | 0.508 |  |

1. Equally credited authors. [↑](#footnote-ref-1)
2. * Corresponding author.

   *E-mail address*: [kondor.attila@csfk.mta.hu](mailto:kondor.attila@csfk.mta.hu) (A. Kondor).

   Phone: +36 1 309-2600 [↑](#footnote-ref-2)
3. * after derivatization [↑](#footnote-ref-3)
4. [↑](#footnote-ref-4)
